# Unravelling the proteomic signature of extracellular vesicles released by drug-resistant *Leishmania infantum* parasites

**DOI:** 10.1371/journal.pntd.0008439

**Published:** 2020-07-06

**Authors:** Noélie Douanne, George Dong, Mélanie Douanne, Martin Olivier, Christopher Fernandez-Prada

**Affiliations:** 1 Department of Pathology and Microbiology, Faculty of Veterinary Medicine, Université de Montréal, Saint-Hyacinthe, QC, Canada; 2 The Research Group on Infectious Diseases in Production Animals (GREMIP), Faculty of Veterinary Medicine, Université de Montréal, Saint-Hyacinthe, QC, Canada; 3 The Research Institute of the McGill University Health Centre, Montréal, QC, Canada; 4 Department of Biology, Health and Ecology, “*Ecole Pratique des Hautes Etudes*”, Paris, France; Universiteit Antwerpen, BELGIUM

## Abstract

Leishmaniasis constitutes the 9^th^ largest disease burden among all infectious diseases. Control of this disease is based on a short list of chemotherapeutic agents headed by pentavalent antimonials, followed by miltefosine and amphotericin B; drugs that are far from ideal due to host toxicity, elevated cost, limited access, and high rates of drug resistance. Knowing that the composition of extracellular vesicles (EVs) can vary according to the state of their parental cell, we hypothesized that EVs released by drug-resistant *Leishmania infantum* parasites could contain unique and differently enriched proteins depending on the drug-resistance mechanisms involved in the survival of their parental cell line. To assess this possibility, we studied EV production, size, morphology, and protein content of three well-characterized drug-resistant *L*. *infantum* cell lines and a wild-type strain. Our results are the first to demonstrate that drug-resistance mechanisms can induce changes in the morphology, size, and distribution of *L*. *infantum* EVs. In addition, we identified *L*. *infantum*’s core EV proteome. This proteome is highly conserved among strains, with the exception of a handful of proteins that are enriched differently depending on the drug responsible for induction of antimicrobial resistance. Furthermore, we obtained the first snapshot of proteins enriched in EVs released by antimony-, miltefosine- and amphotericin-resistant parasites. These include several virulence factors, transcription factors, as well as proteins encoded by drug-resistance genes. This detailed study of *L*. *infantum* EVs sheds new light on the potential roles of EVs in *Leishmania* biology, particularly with respect to the parasite’s survival in stressful conditions. This work outlines a crucial first step towards the discovery of EV-based profiles capable of predicting response to antileishmanial agents.

## Author summary

Visceral leishmaniasis is a life-threatening disease caused by *Leishmania infantum* parasites, which are transmitted by sand flies. In the absence of vaccines, current control of this disease is based on chemotherapy, which is comprised of a very limited arsenal threatened by the emergence and spread of drug-resistant strains. In the shadow of growing concern and treatment failure due to resistance, the characterization of extracellular vesicles (EVs) released by drug-resistant *L*. *infantum* parasites could shed some light on the complex nature of drug resistance in *Leishmania* and increase our understanding of the biology of the parasite. EVs are vesicles secreted by all eukaryotic cells whose contents (proteins, DNA/RNAs, lipids) vary as a function of their cellular origin. Our results demonstrate for the first time that EVs released by drug-resistant parasites are enriched in unique protein markers that reflect the drug-resistance mechanisms involved in the survival of parental cells. These unique proteins included several virulence and transcription factors, as well as drug-resistance genes; this offers a potential benefit for drug-resistant parasites in terms of parasite-to-parasite communication and host-parasite interactions. Collectively, our initial results could serve as a jumping-off point for the future development of novel EV-based diagnostic tools for the detection and appraisal of antimicrobial-resistant *Leishmania* populations.

## Introduction

The vector-borne protozoan parasite *Leishmania* affects 15 million people worldwide, and in the absence of effective preventive and therapeutic treatments, is spreading with ≈1.5M new cases/year [1, 2]. Clinical disease ranges from the self-healing cutaneous manifestation to life-threatening visceral leishmaniasis (VL), a systemic disease with a fatality rate as high as 100% within 2 years, especially if left untreated [3–5]. *L*. *infantum* is the main causative agent of VL in both humans and dogs, the latter of which serve as the main reservoir for infection [6]. While many efforts have been employed to tackle leishmaniasis, the incidence of VL is increasing in the Americas [7], as well as re-emerging in the form of epidemic outbreaks in Europe, India, and Eastern Africa [8–11]. Control of the disease is based on a very short list of chemotherapeutic agents headed by pentavalent antimonials (Sb), followed by miltefosine (MF) and amphotericin B (AmB). These drugs are far from ideal due to host toxicity, elevated cost, and limited access, but in particular due to the high rates of drug resistance [2]. The latter should be not neglected, especially when both humans and dogs are treated with the same molecules [1, 12].

Extracellular vesicles (EVs) are particles formed by a lipid bilayer containing proteins and nucleic acids; EVs originate from and are released by many types of cells, including eukaryotic cells [13–16]. Although EVs can be separated into numerous subclasses, these particles are often categorized as exosomes, microvesicles or apoptotic bodies, according to their size, origin, and components [13, 15, 17]. During EVs biogenesis, different cellular components are loaded into the vesicles, while specific proteins are integrated into their lipid bilayer. Consequently, EVs’ content varies as a function of their cellular origin [18]. EVs have been the focus of numerous studies due to their implication in intercellular communication [19]. There is growing evidence that EVs play a major role not only in parasite-to-parasite communication, but also in host-parasite interactions [20–28]. Of note, it has been determined that *Leishmania* exosomes (a subclass of EVs formed from late endosomes) isolated from *in vitro* cultures are as effective in modulating some early macrophage and host inflammatory responses as whole *Leishmania* parasites [29–31]. Additionally, *Leishmania* exosomes have recently been shown to serve as an envelope and shuttle for complete LRV1 viruses, facilitating virus transmission and increasing infectivity in the mammalian host [32]. Exosomes and other EVs are reported to be involved in cell stress responses and resistance to chemotherapeutic agents in eukaryotic cells [33]. Drug-resistance in *Leishmania* relies on a number of mechanisms, and different key players have been identified in the last decade. For example, *Leishmania* resists Sb by altering its detoxifying pathways, drug uptake, and efflux, and sequestrating Sb-thiol complexes into intracellular vesicles using ABC transporter MRPA [34–36]. Both MF and AmB affect the integrity of different membranes (cell, mitochondria, etc.), and resistance to these drugs induces important changes in specific subsets of the proteome of the parasite [35, 37].

While several studies have demonstrated the utility of whole-parasite proteomics in differentiating sensitive and resistant isolates and in *in vitro*-generated strains [35, 37], the composition of EVs released by drug-resistant *Leishmania* parasites remains unexplored. Knowing that the composition of EVs can vary according to the parental cell [38–40], we hypothesized that EVs released by drug-resistant *L*. *infantum* parasites contain differently enriched proteins that could serve as proxy for the different drug-resistance and compensatory mechanisms deployed by the releasing cell line. To assess this possibility, we used quantitative label-free proteomics to study EVs production, size, morphology, and protein content of three drug-resistant *L*. *infantum* cell lines and a wild-type strain. These experiments lead to the identification of key proteins within EVs issued from drug-resistant parasites, which are essential to understanding how drug-resistant EVs may interact and modulate not only other parasites, but also host cells and the sand fly vector. Moreover, our powerful insights open the door for further exploration of *Leishmania* EV-profiles as potential biomarkers for the identification and evaluation of drug-resistant parasites.

## Methods

### *Leishmania* cultures

The *Leishmania infantum* (MHOM/MA/67/ITMAP-263) wild-type strain (WT) and the *in vitro* generated resistant mutants Sb2000.1, AmB1000.1 and MF200.5 [41–46], which are resistant to 2000 μM of Sb, 1000 nM of AmB and 200 μM MF, respectively, were grown in M199 medium at 25°C supplemented with 10% fetal bovine serum, 5 μg/mL of haemin at pH 7.0 and 2000 μM Sb (Potassium antimonyl tartrate, Sigma-Aldrich), 200 μM of MF (Miltefosine, Cayman Chem.) or 1 μM AmB (Amphotericin B solution, Sigma). Antileishmanial values in promastigotes were determined by monitoring the growth of parasites after 72 h of incubation at 25°C in the presence of increasing antimony concentrations, by measuring A600 using a Cytation 5 machine (BioTek, USA). EC_50_ values were calculated based on dose-response curves analyzed by non-linear regression with GraphPad Prism 8.0 software (GraphPad Software, La Jolla California, USA). An average of at least three independent biological replicates was performed for each determination.

### Purification of *Leishmania* extracellular vesicles

The purification of *Leishmania* EVs was performed as we previously described [29, 32]. Briefly, 1 L of *Leishmania* parasites (2.5–5.0 x 10^7^ parasites/mL) was grown in drug-free M199 at 25°C (supplemented with 10% FBS, 5 μg/mL of haemin at pH 7.0), and left to divide in 10 non-ventilated 75 cm^2^ culture flasks (Corning, USA) until they reached late-log phase. Next, parasites were washed twice in PBS and resuspended in 5 mL RPMI-1640 medium without FBS and phenol red (Life Technologies) at a final concentration of 2.5–5.0 x 10^8^ parasites/mL, in non-ventilated 25 cm^2^ culture flasks (Corning, USA) at 37°C. Parasites were then incubated for 4 h at 37°C to stimulate the release of EVs in the medium [21]. The viability of parasites was evaluated by propidium-iodide staining before and after incubation at 37°C. Only cultures with a viability > 95.0% were submitted to subsequent EV-purification steps. After the 4-h incubation, samples were centrifuged twice to eliminate parasites (10 min at 3 000 *g*) and clear out debris (10 min at 8 500 *g*), followed by two subsequent filtrations using 0.45 μm and 0.20 μm syringe filters. EVs were then recovered by a 1-h centrifugation at 100 000 *g* and resuspended in the EVs buffer (137 mM NaCl, 20 mM Hepes, pH 7.5). EVs’ proteins were then dosed using the Micro BCA Protein Assay Kit (Pierce Biotechnology, USA). EVs were conserved in aliquots in EVs buffer at -80°C for subsequent analyses. Aliquots were slowly thawed on ice before being used. Once thawed, EVs were never refrozen. Three independent purifications were performed for each strain.

### Transmission Electron Microscopy (TEM)

EVs purified from the different *L*. *infantum* WT and drug-resistant strains were directly coated on formvar carbon grids, fixed with 1% glutaraldehyde in 0.1 M sodium cacodylate buffer for 1 min, and stained with 1% uranyl acetate for 1 min. Formvar grids coated with isolated EVs were recorded using a FEI Tecnai 12 120 kV transmission electron microscope. The resulting images were captured with the AMT XR-80C CCD Camera System (Facility for Electron Microscopy Research, McGill University).

### Nanoparticle Tracking Analysis (NTA)

EVs’ concentration and size distribution were characterized by NTA using a NanoSight LM 10 Instrument (Malvern Panalytical, Worcestershire, UK) available in the laboratory of Dr Janusz Rak (RI-MUCH McGill University). To determine the size and number of particles, five sequential 30-s videos were collected using the default parameter settings of the machine. EVs buffer was used as the negative control. The average size and concentration of the particles were calculated by integrating the averages of the modes from three independent records, obtained for each of three independent biological replicates.

### Protein digestion and liquid chromatography–MS/MS (LC-MS/MS)

LC-MS/MS was performed, as previously described [21, 32], at the proteomic platform of the *Institut de Recherches Cliniques de Montréal* (Montréal, Canada). Briefly, proteins derived from purified *Leishmania* EVs were precipitated with 15% TCA/acetone and processed for LC–MS/MS analysis. Protein extracts were then solubilized using a 6M urea buffer, reduced by the addition of reduction buffer (45 mM DTT, 100 mM ammonium bicarbonate) for 30 minutes at 37°C, with a final concentration of 25%, then alkylated by the addition of alkylation buffer (100 mM iodoacetamide, 100 mM ammonium bicarbonate) for 20 minutes at 24°C in the dark, with a final concentration of 25%. Subsequently, an in-solution digestion was performed by the addition of trypsin at a ratio of 1:25 protease/protein, followed by an overnight incubation at 37°C. Finally, formic acid was added to a 0.2% final concentration to quench the reaction. Samples were cleaned with C18 Zip Tip pipette tips (Millipore, USA) before MS analysis. Extracted peptides were injected into a Zorbax Extended-C18 desalting column (Agilent) and separated by chromatography on a Biobasic 18 Integrafrit capillary column (Thermo Scientific, USA) on a Nano high-performance LC system (1100 series unit; Agilent). Eluted peptides were electrosprayed as they exited the capillary column and were analyzed on a QTRAP 4000 linear ion trap mass spectrometer (SCIEX/ABI).

### Protein identification

The peak list files were generated with Distiller version 2.1.0.0 software (www.matrixscience.com/distiller) using the following parameters: minimum mass set to 500 Da, maximum mass set to 6,000 Da, no grouping of MS/MS spectra, precursor charge set to auto, and minimum number of fragment ions set to 5. Protein database searching was performed with Mascot 2.6 (Matrix Science) against the *L*. *infantum* (txid5671, 16,895 proteins) protein database (RefSeq assembly accession: GCF_000002875.2; last access November 2019). The mass tolerances for precursor and fragment ions were set to 10 ppm and 0.6 Da, respectively. Trypsin was used as the enzyme, allowing for up to 1 missed cleavage. Cysteine carbamidomethylation was specified as a fixed modification, and methionine oxidation as variable modifications. Data analysis was performed using Scaffold (version 4.11.0). Peptide identifications were accepted if they could be established with a probability greater than 80.0%. Protein identifications were accepted if they could be established at greater than 95.0% probability and contained at least 2 identified peptides. Protein probabilities were assigned by the Protein Prophet algorithm [47]. Proteins that contained similar peptides and could not be differentiated using MS/MS analysis alone were grouped to satisfy the principles of parsimony. The final number of peptides per protein was represented by the average of the three biological replicates after normalization to the total number of peptides. Normalization of total spectrum counts (TSC) for each protein was performed automatically using Scaffold. Briefly, Scaffold calculates the total number of spectra in each replicate, as well as the average number of spectra across all replicates. The software then multiplies each spectrum count in each sample by the average count over the replicate’s total spectral count, leading to normalized TSC values. Normalized TSC were subsequently used to establish the different comparisons depicted in Tables 1–5.

**Table 1:** Differential enrichment of the common proteins identified in EVs released by *L*. *infantum* WT and the three mutant strains.

Gene ID	Protein	WT	Sb2000.1	MF200.5	AmB1000.1
LINJ_26_0150	60S ribosomal protein L7 putative	93.3	77.0 (0.83)	46.7 (0.50)	5.7 (0.06)
LINJ_36_4100	Adenosyl homocysteinase	13.7	7.7 (0.56)	12.7 (0.93)	7.0 (0.51)
LINJ_32_0410	ATP-dependent RNA helicase putative	95.7	49.0 (0.51)	89.7 (0.94)	9.7 (0.10)
LINJ_17_0180	Elongation factor 1-alpha	75.3	65.7 (0.87)	115.7 (1.54)	47.0 (0.62)
LINJ_17_0190	Elongation factor 1-alpha	112.3	92.0 (0.82)	148.3 (1.32)	66.3 (0.59)
LINJ_36_0190	Elongation factor 2	21.3	5.0 (0.23)	16.7 (0.78)	6.7 (0.31)
LINJ_14_1240	Enolase	37.0	43.3 (1.17)	45.0 (1.22)	21.7 (0.59)
LINJ_10_0520	GP63-leishmanolysin	24.7	14.3 (0.58)*	53.0 (2.15)	9.7 (0.39)*
LINJ_10_0490	GP63-leishmanolysin	113.3	65.7 (0.58)**	243.3 (2.15)	66.7 (0.59)*
LINJ_10_0800	GP63-leishmanolysin	58.7	34.0 (0.58)*	163.3 (2.78)	42.3 (0.72)
LINJ_10_0530	GP63-leishmanolysin	74.7	45.0 (0.60)*	123.3 (1.65)	49.0 (0.66)
LINJ_33_0350	Heat shock protein 83–1	36.0	37.7 (1.05)	49.7 (1.38)	21.3 (0.59)
LINJ_28_3000	Heat shock protein (Fragment)	73.7	61.3 (0.83)	85.0 (1.15)	41.0 (0.56)
LINJ_26_1220	Heatshock70-related protein 1 mitochondrial precursor putative	14.7	18.7 (1.27)	18.7 (1.27)	12.0 (0.82)
LINJ_18_1350	Heatshock protein 110 putative	18.7	15.3 (0.82)	31.3 (1.68)	5.0 (0.27)
LINJ_15_0010	Histone H4	13.0	15.3 (1.18)	24.0 (1.85)	5.3 (0.41)
LINJ_32_3100	Nucleoside diphosphate kinase	14.7	8.3 (0.57)*	19.0 (1.30)	7.3 (0.50)**
LINJ_09_0950	Polyubiquitin	13.3	8.7 (0.65)	7.7 (0.58)	6.3 (0.48)
LINJ_04_0750	Putative 60S ribosomal protein L10	86.7	70.7 (0.82)	46.3 (0.53)	14.3 (0.17)
LINJ_16_0470	Putative 60S ribosomal protein L21	42.3	35.7 (0.84)	44.0 (1.04)	6.7 (0.16)
LINJ_34_3440	Putative 60S ribosomal protein L21	42.0	36.7 (0.87)	44.3 (1.06)	6.7 (0.16)
LINJ_27_2350	Putative heat shock protein DNAJ	9.3	4.7 (0.50)	10.3 (1.11)	4.0 (0.43)
LINJ_28_3060	Putative heat shock protein hsp70	90.3	72.7 (0.80)	92.3 (1.02)	48.7 (0.54)
LINJ_27_0620	Putative small GTP-binding protein Rab1	9.3	3.7 (0.39)	9.3 (1.00)	4.3 (0.46)
LINJ_31_1240	Pyrophosphate-energized vacuolar membrane proton pump1 putative	36.3	9.3 (0.26)	47.0 (1.24)	10.7 (0.29)
LINJ_35_5450	Pyruvate kinase	10.7	6.0 (0.56)	10.0 (0.94)	11.0 (1.03)
LINJ_36_1420	Transitional endoplasmic reticulum ATPase putative	12.7	5.7 (0.45)	11.3 (0.89)	4.3 (0.34)
LINJ_13_1450	Tubulin alpha chain	52.7	44.7 (0.85)	91.3 (1.73)*	25.7 (0.49)
LINJ_08_1290	Tubulin beta chain	46.3	39.0 (0.84)	130.3 (2.81)*	28.3 (0.61)
LINJ_08_1290	Tubulin beta chain	45.0	37.0 (0.82)	129.7 (2.88)*	29.3 (0.65)
LINJ_26_1960	Uncharacterized protein	13.7	14.0 (1.02)	18.3 (1.34)	12.7 (0.93)

Numbers correspond to average Total Spectrum Count (n = 3). Numbers between parentheses depict the average fold change of the mutant compared with the WT.

* *p* < *0*.*05*

** *p* < *0*.*01* (unpaired t-test).

**Table 2:** Enriched and unique proteins identified in EVs isolated from the WT *L*. *infantum* strain.

	Gene ID	Protein	Total Spectrum Count^1^	(Trans)membrane protein	Unconventional secretion prediction^2^	Signal peptide^3^
1	LINJ_31_0450	Putative cytoskeleton associated protein CAP5.5	21.3	no	no (0.465)	no
2	LINJ_31_0820	C2 domain protein—putative	8.3	no	no (0.154)	no
3	LINJ_31_1860	Aminoacid permease	8.0	yes	yes (0.662)	no
4	LINJ_13_1500	Programmed cell death 6 protein-like protein	7.7	no	yes (0.937)	no
5	LINJ_35_2080	Putative calcium motive P-type ATPase	7.7	yes	yes (0.612)	no
6	LINJ_12_0668*	Surface antigen protein 2-putative	7.3	yes	no (0.295)	yes
7	LINJ_12_0490	Glucose-6-phosphate isomerase	7.0	no	no (0.452)	no
8	LINJ_31_2670	Putative calreticulin	7.0	no	no (0.498)	yes
9	LINJ_19_1280	SPFH domain / Band 7 family—putative	4.7	no	no (0.526)	no
10	LINJ_06_1360	CLN3 protein putative	4.3	yes	no (0.280)	no
11	LINJ_28_2050	Zinc transporter 3-putative	3.7	yes	no (0.514)	yes
12	LINJ_16_1450	ADP-ribosylationfactor-like-putative	3.3	no	no (0.342)	no
13	LINJ_06_1330*	Coproporphyrinogen III oxidase	3.3	no	no (0.556)	no
14	LINJ_31_0810*	Putative c2 domain protein	3.3	no	no (0.336)	no
15	LINJ_29_2610	Vacuolar protein sorting ssociated protein 4	3.3	no	no (0.387)	no
16	LINJ_36_2340	Plasma-membrane choline transporter putative	3.0	yes	yes (0.863)	no
17	LINJ_10_0380	Pteridine transporter putative	3.0	yes	no (0.361)	no
18	LINJ_09_0960	Putative ef-hand protein 5	3.0	no	no (0.311)	no
19	LINJ_36_1680	Putative universal minicircle sequence binding protein	3.0	no	yes (0.822)	no
20	LINJ_14_0490	Amastin surface glycoprotein putative	2.3	yes	yes (0.838)	no
21	LINJ_31_0050*	BT1 family MFS sugar transport protein putative	2.3	yes	no (0.466)	no
22	LINJ_03_0190	Delta-1-pyrroline-5-carboxylate dehydrogenase putative	2.3	no	no (0.559)	no
23	LINJ_27_2520	Putative cysteine peptidase, Clan CA, family C2	1.3	no	sequence too long to be analyzed	
Total number of proteins				9 (39.1%)	5 (26.0%)	3 (13.0%)

^1^ Mean Total Spectrum Count obtained with the LC–MS/MS analyses.

^2^ NM score calculated using the SecretomeP 2.0 Server. Proteins with an NM score higher than 0.600 that are not predicted to have a signal peptide are considered non-classically secreted proteins.

^3^Signal peptides were predicted using SignalP Server integrated in SecretomeP 2.0 Server.

*Unique protein specific to EVs released by this strain.

**Table 3:** Enriched proteins identified in EVs isolated from the Sb2000.1 *L*. *infantum* strain.

	Gene ID	Protein	Total Spectrum Count^1^	(Trans)membrane protein	Unconventional secretion prediction^2^	Signal peptide^3^
1	LINJ_07_0960	Hypothetical protein conserved	17.3	yes	0.693, but prediction of a peptide signal	yes
2	LINJ_30_3650	40S ribosomal protein S14	15.3	no	yes (0.693)	no
3	LINJ_28_1050	40S ribosomal protein S14	15.3	no	yes (0.695)	no
4	LINJ_13_1510	SPRYdomain/HECT-domain (Ubiquitin-transferase) putative	14.3	no	sequence too long to be analyzed	
5	LinJ_15_0540	Ecotin-like protein 3	10.0	no	no (0.343)	no
6	LINJ_33_2070	Ribosomal protein L37	6.7	no	yes (0.783)	no
7	LINJ_34_3420	Uncharacterized protein	5.7	yes	0.825, but prediction of a peptide signal	yes
8	LINJ_16_0510	Hypothetical protein conserved	5.3	yes	no (0.126)	yes
9	LINJ_02_0340	Putative proteasome regulatory non-ATPase subunit 6	4.7	no	no (0.097)	no
10	LINJ_27_0500	Putative calpain-like cysteine peptidase	4.0	no	sequence too long to be analyzed	
11	LINJ_19_0560	Hypothetical protein conserved	3.7	yes	0.653, but prediction of a peptide signal	yes
12	LINJ_22_0440	Putative proteasome regulatory ATPase subunit 1	3.7	no	yes (0.659)	no
13	LINJ_28_0090	Adenylate cyclase-like protein	3.3	no	no (0.358)	no
14	LINJ_20_1310	Domain of unknown function (DUF1935)—putative	3.3	no	no (0.599)	no
15	LINJ_21_0830	Hypothetical protein conserved	3.0	no	no (0.402)	no
16	LINJ_28_3090	Malate dehydrogenase	3.0	no	no (0.447)	no
17	LINJ_16_1550	Putative kinesin	2.7	no	no (0.394)	no
18	LINJ_32_4040	Cysteine peptidase—putative	2.3	no	yes (0.620)	no
19	LINJ_30_1710	SmallGTP-binding protein Rab28 putative	2.3	no	no (0.450)	no
20	LINJ_01_0010	Protein of unknown function (DUF2946)	2.0	yes	no (0.085)	yes
21	LINJ_29_2550	Phosphodiesterase putative	1.7	no	no (0.354)	no
22	LINJ_25_1640	Putative casein kinase I	1.3	no	no (0.559)	no
23	LINJ_31_1130	Putative N-acyl-L-amino acid amidohydrolase	1.3	no	no (0.530)	no
24	LINJ_32_2610	Hypothetical protein conserved	1.3	no	no (0.408)	no
25	LINJ_20_0160	Hypothetical protein conserved	1.0	no	no (0.080)	no
Total number of proteins				5 (20.0%)	5 (20.0%)	5 (20.0%)

^1^ Mean Total Spectrum Count obtained with the LC–MS/MS analyses.

^2^ NM score calculated using the SecretomeP 2.0 Server. Proteins with an NM score higher than 0.600 that are not predicted to have a signal peptide are considered non-classically secreted proteins.

^3^Signal peptides were predicted using SignalP Server integrated in SecretomeP 2.0 Sever.

**Table 4:** Enriched and unique proteins identified in EVs isolated from the MF200.5 *L*. *infantum* strain.

	Gene ID	Protein	Total Spectrum Count^1^	(Trans)membrane protein	Unconventional secretion prediction^2^	Signal peptide^3^
1	LINJ_29_2620	ATP-dependent 6-phosphofructokinase	69.3	no	no (0.295)	no
2	LINJ_27_1710	Glycosomal phosphoenol pyruvate carboxykinase, putative	66.3	no	no (0.279)	no
3	LINJ_21_0310	Phosphotransferase	52.0	no	no (0.454)	no
4	LINJ_11_1000	Pyruvate, phosphate dikinase	34.3	no	no (0.442)	no
5	LINJ_36_1320	Fructose-bisphosphate aldolase	32.0	no	no (0.547)	no
6	LINJ_35_1190	Putative NADH-dependent fumarate reductase	30.3	no	no (0.285)	no
7	LINJ_02_0430	Voltage-dependent anion-selective channel—putative	30.0	no	no (0.550)	no
8	LINJ_30_2480	Heatshock70-related protein1 -mitochondrial precursor putative	28.0	no	no (0.234)	no
9	LINJ_36_7320	Putative eukaryotic translation initiation factor 3 subunit 8	26.0	no	no (0.129)	no
10	LINJ_16_0560	Orotidine 5 phosphate decarboxylase/orotate phosphoribosyl transferase putative	19.0	no	yes (0.850)	no
11	LINJ_35_3150	ATP-dependent RNA helicase putative	18.0	no	no (0.171)	no
12	LINJ_17_0870	GMP reductase	17.7	no	yes (0.655)	no
13	LINJ_29_2310	Dynamin-1-like protein	14.7	no	no (0.454)	no
14	LINJ_36_0270	Eukaryotic translation initiation factor 3 subunit L	14.3	no	no (0.259)	no
15	LINJ_17_0010	Hypothetical protein conserved	14.3	no	no (0.309)	no
16	LINJ_36_7070	ATP synthase delta (OSCP) subunit putative	12.0	no	yes (0.807)	no
17	LINJ_32_3470	Chaperonin alpha subunit-putative	11.3	no	no (0.414)	no
18	LINJ_17_1390	Translation initiation factor putative	11.3	no	no (0.266)	no
19	LINJ_35_3750	Putative Gim5A protein	10.3	no	yes (0.705)	no
20	LINJ_18_0510	Aconitate hydratase	10.0	no	no (0.493)	no
21	LINJ_30_3430	Phosphoglycerate kinase	10.0	no	no (0.501)	no
22	LINJ_30_0120	Alkyl dihydroxy acetone phosphate synthase O	9.3	no	no (0.400)	no
23	LINJ_32_3200	Leucine-rich repeat protein-putative	9.3	no	no (0.568)	no
24	LINJ_22_1390	Alanine—tRNA ligase	9.0	no	no (0.324)	no
25	LINJ_36_1000	40S ribosomal protein S18-putative	8.3	no	no (0.667)	no
26	LINJ_10_0070	Dehydrogenase like protein	8.3	yes	0.820, but prediction of a peptide signal	yes
27	LINJ_23_0050	Peroxidoxin	8.3	no	yes (0.854)	no
28	LINJ_24_2150	Transketolase	8.3	no	no (0.569)	no
29	LINJ_35_1490	Arginase	8.0	no	no (0.376)	no
30	LINJ_29_0120	Proteasome regulatory non-ATPase subunit putative	7.7	no	no (0.519)	no
31	LINJ_07_0710	Hypothetical protein conserved	7.3	no	yes (0.609)	no
32	LINJ_05_0280	Protein tyrosine phosphatase putative	7.3	yes	no (0.100)	no
33	LINJ_32_2280	Hypothetical protein conserved	7.0	no	yes (0.628)	no
34	LINJ_11_0100	Seryl-tRNA synthetase putative	7.0	no	no (0.330)	no
35	LINJ_24_0870	Triosephosphate isomerase	7.0	no	no (0.413)	no
36	LINJ_23_0810	Uncharacterized protein	7.0	yes	no (0.254)	yes
37	LINJ_30_3080	Eukaryotic translation initiation factor 3 subunit 7-like protein	6.7	no	no (0.196)	no
38	LINJ_19_1590	Inosine-5'-monophosphate dehydrogenase	6.7	no	no (0.331)	no
39	LINJ_25_2520	Uncharacterized protein	6.7	no	no (0.281)	no
40	LINJ_35_1000	Aldose 1 epimerase putative	6.3	no	no (0.598)	no
41	LINJ_32_3510	Dihydrolipoyl dehydrogenase	6.3	no	no (0.463)	no
42	LINJ_36_3220	Fibrillarin	6.0	no	no (0.334)	no
43	LINJ_28_1830	Hypothetical protein conserved	6.0	no	no (0.129)	no
44	LINJ_29_2300	Hypothetical protein conserved	6.0	yes	0.675, but prediction of a peptide signal	yes
45	LINJ_19_1560	Peptidylprolyl isomerase	6.0	no	yes (0.665)	no
46	LINJ.20.0120	Phosphoglycerate kinase	6.0	no	no (0.226)	no
47	LINJ_11_1100	Sterol14-alpha-demethylase putative	6.0	no	0.829, but prediction of a peptide signal	yes
48	LINJ_36_3940	40S ribosomal protein S27-1 putative	5.7	no	yes (0.681)	no
49	LINJ_27_1770	Trypanothione synthetase putative	5.7	no	no (0.415)	no
50	LINJ_19_1100	Proteasome regulatory non-ATPase subunit9 putative	5.3	no	no (0.532)	no
51	LINJ_36_4030	Putative glycyl tRNA synthetase	5.3	no	no (0.436)	no
52	LINJ_34_0010	Uncharacterized protein	5.3	no	yes (0.738)	no
53	LINJ_32_3460	Glucosamine-6-phosphate isomerase	5.0	no	yes (0.835)	no
54	LINJ_36_6080	Nitroreductase family putative	5.0	no	no (0.511)	no
55	LINJ_36_7280	Protein disulfide-isomerase	5.0	no	0.649, but prediction of a peptide signal	yes
56	LINJ_35_0370	ATP-dependent DEAD-box RNA helicase putative	4.7	no	no (0.407)	no
57	LINJ_32_1060	Chaperon incontaining t-complex protein putative	4.7	no	no (0.407)	no
58	LINJ_15_0270	Lysine-tRNA ligase	4.7	no	no (0.337)	no
59	LINJ_22_0310	40S ribosomal protein S15 putative	4.3	no	no (0.453)	no
60	LINJ_10_0560*	Glycerol-3-phosphate dehydrogenase [NAD(+)]	4.3	no	yes (0.633)	no
61	LINJ_27_1150	Putative T-complex protein 1, beta subunit	4.3	no	no (0.393)	no
62	LINJ_30_3480*	Protein_mkt1_-_putative	4.3	no	no (0.505)	no
63	LINJ_36_4380	Zeta-crystallin/NADPH-oxidoreductase-like protein	4.3	no	yes (0.670)	no
64	LINJ_27_1230	Arginyl-tRNA synthetase-putative	4.0	no	no (0.393)	no
65	LINJ_27_2020	RNA-binding protein putative	4.0	no	no (0.378)	no
66	LINJ_36_3750	Cysteine synthase	3.7	no	no (0.411)	no
67	LINJ_36_4070	Eukaryotic translation initiation factor 3 subunit I	3.7	no	no (0.405)	no
68	LINJ_02_0330	Putative casein kinase II, alpha chain	3.7	no	no (0.598)	no
69	LINJ_28_2480	Eukaryotic translation initiation factor 3 subunit E	3.3	no	no (0.557)	no
70	LINJ_25_1670	Hypothetical protein conserved	3.3	no	no (0.451)	no
71	LINJ_36_2510	Methyltransferase	3.3	no	no (0.339)	no
72	LINJ_29_2270	Rab GDP dissociation inhibitor	3.3	no	no (0.541)	no
73	LINJ_36_5330	Hypothetical protein conserved	3.3	no	no (0.344)	no
74	LINJ_06_0120	Peptidyl-prolyl cis-trans isomerase	3.0	no	0.842, but prediction of a peptide signal	yes
75	LINJ_03_0670	DEAD/DEAH box helicase /Type III restriction enzyme res subunit—putative	3.0	no	no (0.324)	no
76	LINJ_19_0090	Fibrillarin putative	2.7	no	yes (0.659)	no
77	LINJ_18_0270	Glycogen synthase kinase 3 putative	2.7	no	no (0.499)	no
78	LINJ_11_0640	Putative aminopeptidase	2.7	no	no (0.499)	no
79	LINJ_10_0210	Putative nucleolar protein	2.7	no	no (0.396)	no
80	LINJ_29_2680	Putative serine/threonine-protein kinase	2.7	no	yes (0.613)	no
81	LINJ_32_2330	SAC3/GANP/Nin1/mts3/eIF-3 p25 family /COP9 signalosome—subunit CSN8—putative	2.7	no	yes (0.850)	no
82	LINJ_29_2350	Aminopeptidase	2.3	no	yes (0.606)	no
83	LINJ_36_6170	Halo acid dehalogenase-like hydrolase putative	2.3	no	0.714, but prediction of a peptide signal	yes
84	LINJ_18_0670	HEAT repeats/HEAT repeat putative	2.3	no	no (0.439)	no
85	LINJ_09_1130	Translation initiation factor EIF-2B gamma subunit putative	2.3	no	yes (0.655)	no
86	LINJ_35_2400	Aminopeptidase P putative	2.0	no	yes (0.680)	no
87	LINJ_08_0960	Cysteine peptidase B (CPB)	2.0	no	0.790, but prediction of a peptide signal	yes
88	LINJ_34_3160*	Glucose-6-phosphate 1-epimerase	2.0	no	yes (0.606)	no
89	LINJ_28_3050	Hypothetical protein conserved	2.0	no	no (0.183)	no
90	LINJ_35_2090	Kinesin putative	2.0	no	no (0.548)	no
91	LINJ_03_0240	Ribosomal protein L38 putative	2.0	no	yes (0.723)	no
92	LINJ_15_1170*	Cyclic nucleotide-binding domain containing protein—putative	2.0	no	no (0.367)	no
93	LINJ_27_1300	60S acidic ribosomal protein P0	1.7	no	no (0.208)	no
94	LINJ_21_0980	Hypoxanthine phosphoribosyl transferase	1.7	no	no (0.587)	no
95	LINJ_36_1390	N-acetyltransferase subunit Nat1 putative	1.7	no	no (0.346)	no
96	LINJ_31_3080	Acetyl-CoA carboxylase putative	1.3	no	no (0.316)	no
97	LINJ_35_0840	Aspartate aminotransferase putative	1.3	no	no (0.587)	no
98	LINJ_31_0890	Ras-like small GTPases putative	1.3	no	no (0.208)	no
99	LINJ_27_1920	GMP-PDE-delta subunit-putative/Pfam:PF05351	1.0	no	yes (0.664)	no
Total number of proteins				4 (4.0%)	22 (21.8%)	8 (7.9%)

^1^ Mean Total Spectrum Count obtained with the LC–MS/MS analyses.

^2^ NM score calculated using the SecretomeP 2.0 Server. Proteins with an NM score higher than 0.600 that are not predicted to have a signal peptide are considered non-classically secreted proteins.

^3^Signal peptides were predicted using SignalP Server integrated in SecretomeP 2.0 Sever.

*Unique protein specific to EVs released by this strain.

**Table 5:** Enriched and unique proteins identified in EVs isolated from the AmB1000.1 *L*. *infantum* strain.

	Gene ID	Protein	Total Spectrum Count^1^	(Trans)membrane protein	Unconventional secretion prediction^2^	Signal peptide^3^
1	LINJ_27_1630*	Hypothetical protein conserved	37.7	no	no (0.522)	no
2	LINJ_04_1250*	Actin	15.3	no	no (0.513)	no
3	LINJ_29_0110	C2 domain in Dock180 and Zizimin proteins putative	14.0	no	no (0.272)	no
4	LINJ_31_0520*	Cytoskeleton-associated protein	9.3	no	no (0.465)	no
5	LINJ_26_2510	Hypothetical protein conserved	9.0	no	no (0.107)	no
6	LINJ_05_0580*	Uncharacterized protein	8.0	yes	no (0.196)	no
7	LINJ_13_0090*	Putative carboxypeptidase	7.3	no	no (0.384)	no
8	LINJ_23_0410*	Putative NADP dependent alcohol dehydrogenase	7.3	no	no (0.303)	no
9	LINJ_35_1030*	Putative casein kinase	7.0	no	no (0.343)	no
10	LINJ_31_2890*	ADP-ribosylation factor putative	5.7	no	no (0.512)	no
11	LINJ_34_1630*	p25-alpha putative	5.7	no	yes (0.617)	no
12	LINJ_36_0080*	Stress-inducible protein STI1 homolog	4.7	no	yes (0.645)	no
13	LINJ_19_0250*	Kinesin-like protein	4.3	no	no (0.136)	no
14	LINJ_27_1770*	Putative trypanothione synthetase	3.7	no	no (0.417)	no
15	LINJ_23_1200*	Hydrophilic acylated surface protein a (HASPA1)	2.7	no	no (0.283)	no
16	LINJ_25_2100	Hypothetical protein conserved	2.7	no	no (0.487)	no
17	LINJ_36_3360	14-3-3 protein-like protein	2.3	no	no (0.240)	no
18	LINJ_26_1480	Hypothetical protein conserved	1.7	no	yes (0.867)	no
19	LINJ_23_0290	Pentamidine resistance protein 1	1.7	yes	no (0.497)	no
Total number of proteins				2 (10.5%)	3 (15.8%)	0 (0.0%)

^1^ Mean Total Spectrum Count obtained with the LC–MS/MS analyses.

^2^ NM score calculated using the SecretomeP 2.0 Server. Proteins with an NM score higher than 0.600 that are not predicted to have a signal peptide are considered non-classically secreted proteins.

^3^Signal peptides were predicted using SignalP Server integrated in SecretomeP 2.0 Sever.

*Unique protein specific to EVs released by this strain.

### Bioinformatics analysis

Shared proteins among the three replicates of each strain (WT: 152; Sb2000.1: 194; MF200.5: 264; and AmB1000.1: 70 proteins) were annotated with GO terms from goa_uniprot_all.gaf (UniProt GOA knowledge base; downloaded 12-Nov-2019) using the integrated functions of Scaffold version 4.11.0. Protein information was retrieved from UniprotKB (*L*. *infantum* reference proteome; last modified January 15, 2020) [48] and TriTrypDB v46 (last modified November 6, 2019) [49]. Presence of membrane/transmembrane domains was evaluated for retained proteins using UniprotKB (https://www.uniprot.org/). To this end, Gene IDs (Tables 2–5) were used to retrieve the summary page of each individual protein (last access November 2019), which includes the subcellular localization of the query protein predicted *via* InterPro [50]. Venn diagrams were constructed using the online tool http://bioinformatics.psb.ugent.be/webtools/Venn/. Heat maps of total spectral counts for the common proteins between the WT and the resistant lines were generated using the Heatmapper server [51]. Prediction of non-classical protein secretion was investigated using the SecretomeP 2.0 server (http://www.cbs.dtu.dk/services/SecretomeP/; last access May 2020) [52]. Briefly, the sequence of each protein was recovered from TriTrypDB v46 (https://tritrypdb.org/tritrypdb/) using its unique Gene ID (Tables 2–5). Sequences were assembled in a bulk FASTA file and submitted to SecretomeP 2.0 server. For each input sequence, the server predicted the possibility of non-classical secretion (NN-score). Moreover, SecretomeP 2.0 server integrates SignalP5.0 [53], which allowed us to determine the potential presence of signal peptides on the different proteins. For eukaryotic organisms, proteins with an NN score > 0.600 that are not predicted to have a signal peptide are considered non-classically secreted proteins.

### Statistical analyses of data

Statistical analyses were performed using the unpaired Student’s t-test (*p ≤ 0.*05*, **p ≤ 0.*01*) and Shapiro-Wilk Test for normality, followed by Kruskal-Wallis rank test (****p ≤ 0.*0001*) and post-hoc Nemenyi (Tukey and Kramer) test (**p ≤ 0.*01*, ****p ≤ 0.*0001*). The data were analyzed using GraphPad Prism 8.0 software (GraphPad Software, La Jolla California, USA) and R 3.6.1 software [54].

## Results

### Drug resistance affects *Leishmania* EVs’ quantity, size and distribution

Composition and quantity of EVs released by eukaryotic cells is known to vary according to cell stress conditions (pH, temperature, etc.) and metabolic state [38–40]. To establish whether the capacity to produce and release EVs (including their size and distribution) is altered in drug-resistant *Leishmania* parasites, we selected four well-characterized strains: *L*. *infantum* WT and drug-resistant mutants Sb2000.1 (antimony), AmB1000.1 (amphotericin B), and MF200.5 (miltefosine). Before proceeding to purification of EVs, the four strains were analyzed in terms of drug sensitivity to confirm their previously reported phenotypes [41–46]. As expected, the WT was sensitive to all three drugs, while all mutants were highly resistant to their respective selection drug (S1 Table). EVs were extracted and purified from temperature-stressed cultures of stationary WT and drug-resistant parasites through multiple centrifugation, wash, and filtration steps (as detailed in the materials and methods section). It is important to note that while drug-resistant cultures were maintained under drug pressure, this pressure was removed during EVs production and purification to avoid direct effects of the drug on the membrane of the parasites (*e*.*g*. MF and AmB).

**Fig 1:**
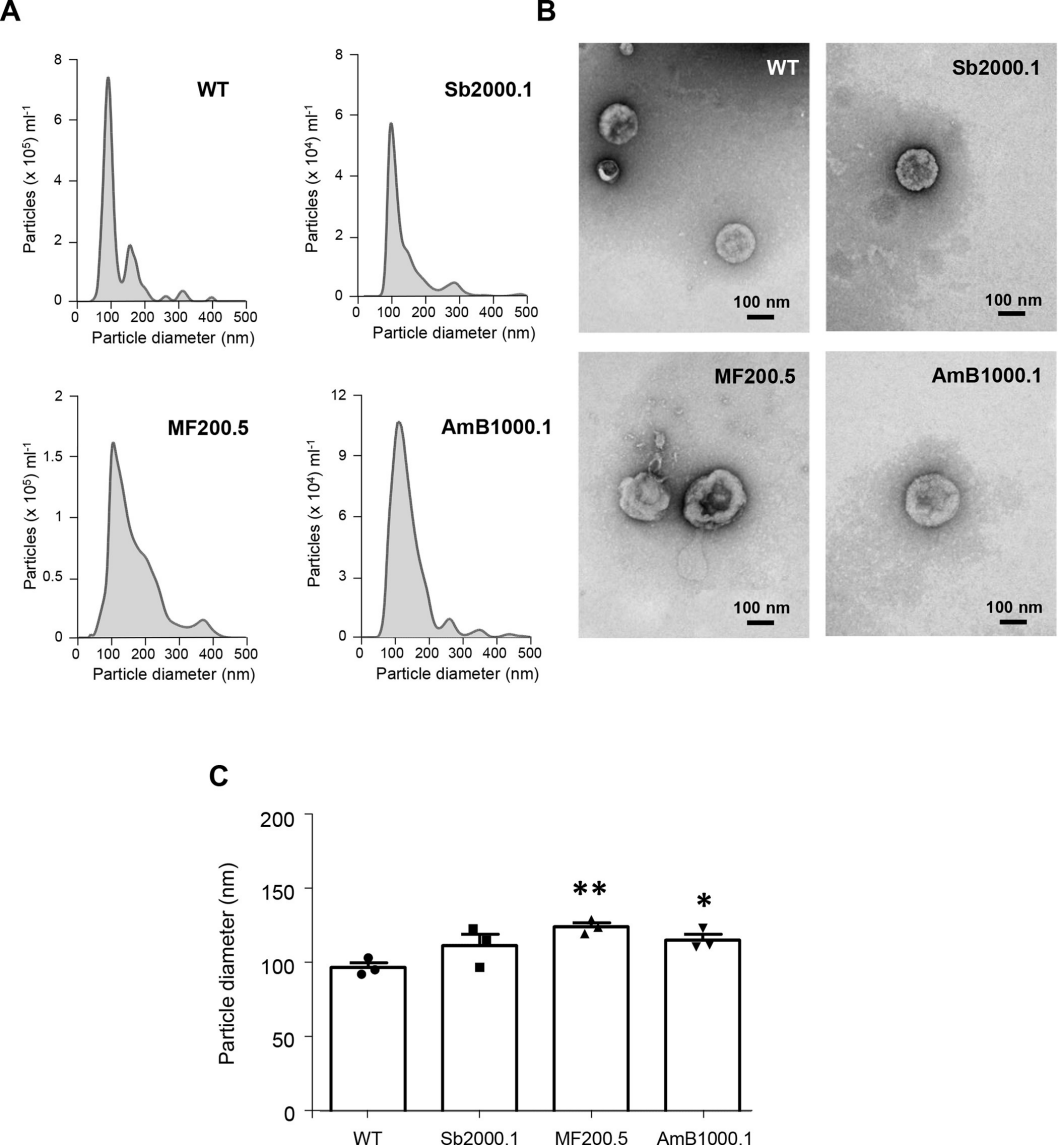
EVs released by *L*. *infantum* parasites differ in morphology and size depending on their drug-resistance background. **(A)** Particle size distributions obtained by nanoparticle tracking analysis of *L*. *infantum* WT, and drug resistant Sb2000.1, MF200.5 and AmB1000.1 strains. Results were normalized per 10^6^ parasites. **(B)** EVs derived from *L*. *infantum* WT, and drug resistant Sb2000.1, MF200.5 and AmB1000.1 promastigotes were prepared for TEM by negative staining. **(C)** Comparison of the modal average size of the EVs isolated from WT, Sb2000.1, MF200.5 and AmB1000.1 promastigotes. Differences were statistically evaluated by unpaired t-test (*p ≤ 0.*05*, (**p ≤ 0.*01*). In a–c, the results are representative of at least three independent experiments with similar data.

Next, purified vesicles were submitted to three nanoparticle-tracking analyses (NTA) per biological replicate to accurately determine the distribution and concentration of the particles (Fig 1A). Following MISEV2018 recommendations [55], NTA analyses and total protein quantification (microBCA) were used to calculate the particle/μg protein ratio for the different EVs’ purifications. All ratios ranged from 10^9^ to 10^10^, with mean values of 2.51 x 10^10^ for the WT, 7.69 x 10^9^ for Sb2000.1, 1.57 x 10^10^ for MF200.5 and 2.98 x10^10^ for AmB1000.1. No significant differences were observed among the strains.

EVs were also evaluated by transmission electron microscopy (Fig 1B), confirming the predominant presence of lipid bilayer-enclosed nano-sized structures compatible with exosomes (~50 to 200 nm in diameter) and other small EVs. NTA and microscopy analyses revealed probable differences in size and morphology in EVs released by drug-resistant strains. To determine the average size of EVs released by the different strains, the modes of each distribution were determined (Sb2000.1: 112.4 nm; MF200.5: 125.0 nm and AmB1000.1: 116.2 nm) and compared with the WT (97.7 nm) (Fig 1C). While all three resistant strains showed a tendency to release larger EVs, only EVs released by MF200.5 and AmB1000.1 were significantly larger (unpaired t-test; *p ≤ 0*.*01* and *p ≤ 0*.*05*, respectively).

**Fig 2:**
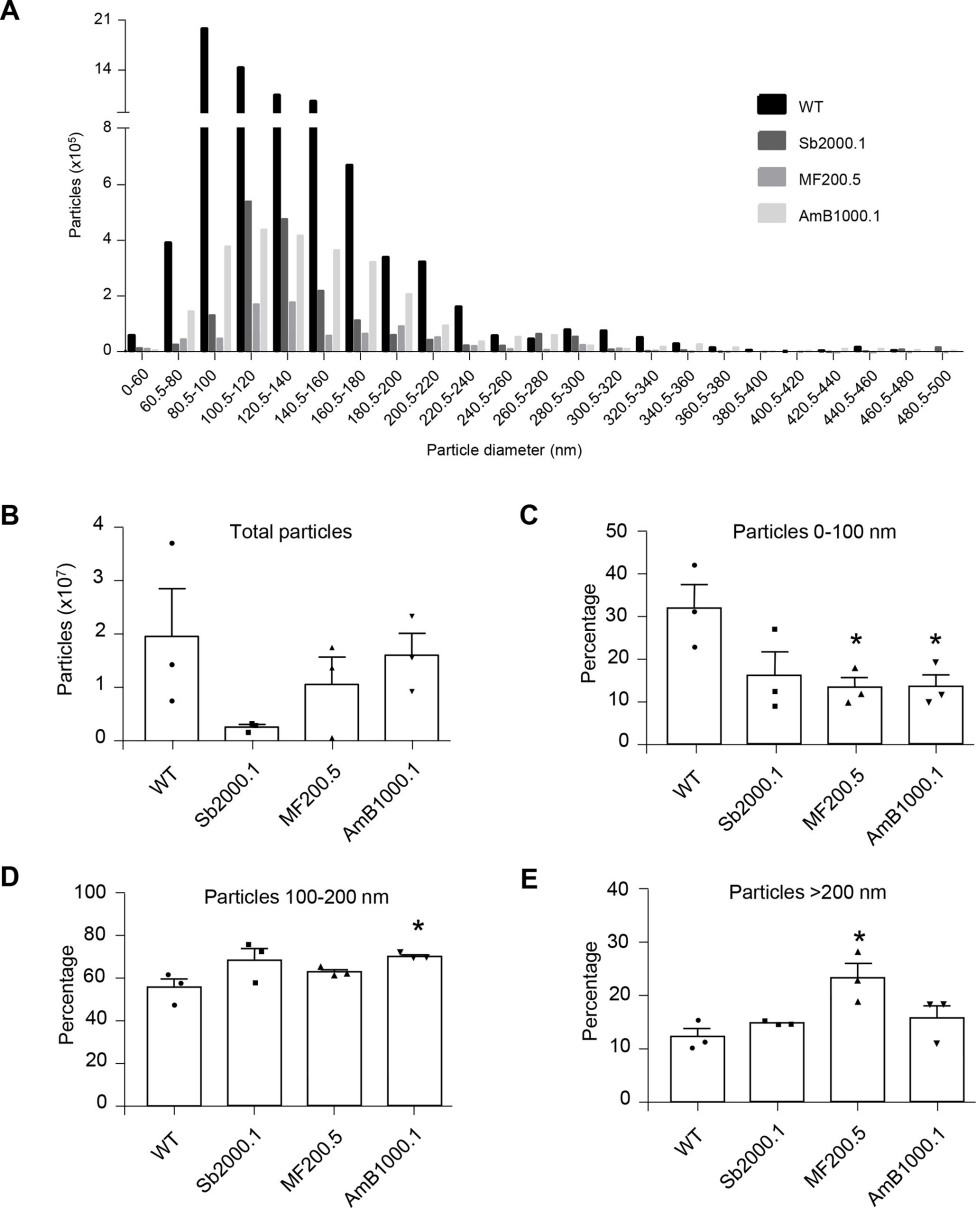
EVs released by drug-resistant *L*. *infantum* parasites show altered particle profiles and EV secretion capacity. **(A)** *L*. *infantum* WT and drug-resistant strains were compared in terms of number of particles per category (size) between 0 nm and 500 nm using NTA. One representative image of three experiments is shown (experiments 2 and 3 are shown in S1). **(B)** Total number of particles, from 0 nm to 500 nm, secreted by WT, Sb2000.1, MF200.5 and AmB1000.1. **(C)** Percentage of particles corresponding to 0–100 nm. **(D)** Percentage of particles corresponding to 100–200 nm. **(E)** Percentage of particles corresponding to 200–500 nm. In A–E, the results are representative of at least three independent experiments with similar data. Differences were statistically evaluated by unpaired t-test (*p < 0.*05*).

The next step was to evaluate if the shift in terms of average size of EVs was affecting the global distribution, or rather specific subpopulations, of said particles. To this end, the NTA datasets corresponding to sizes ranging from 0 to 500 nm, previously identified in Fig 1A, were further analyzed. As depicted in Fig 2A (S1 Fig), the number of particles secreted per size seemed to follow a different trend depending on the strain. To verify this, the global distribution of particles released by the WT and the drug-resistant strains were compared (S2 Fig), revealing significant differences among the four strains (S2 Fig; Kruskal-Wallis rank test; *p ≤ 0*.*01*). Nemenyi post-hoc test (S2 Fig) pinpointed significant differences between WT and Sb2000.1 EV distributions (*p ≤ 0*.*0001*), as well as between Sb2000.1 and the other two resistant strains (*p ≤ 0*.*01* for Sb2000.1 *vs*. MF200.5; *p ≤ 0*.*0001* for Sb2000.1 *vs*. AmB1000.1).

Different quantitative analyses using NTA datasets were performed to verify the likelihood of a direct link between drug resistance and the capacity of *Leishmania* parasites to release different EV subpopulations. For all these comparisons, the number of particles was normalized to 10^6^ parasites. Strain Sb2000.1 was the only cell line showing a strong tendency toward reduced EV secretion. However, this difference was not significant when compared with the WT (Fig 2B; *p = 0*.*065*). As depicted in Fig 2C, particles < 100 nm accounted for approximately 32.0% of the total particles produced by the WT strain. The percentage of particles corresponding to this range was significantly decreased (approximately 13.0% in both cases, *p ≤ 0*.*05*) in MF200.5- and AmB1000.1-resistant strains. While not statistically significant (*p = 0*.*057*), Sb2000.1 showed a tendency to produce less particles < 100 nm. Particles spanning from 100 to 200 nm represented approximately 55–70.0% of all the particles secreted by the four strains analyzed (Fig 2D). AmB1000.1 showed a significant increase in the percentage of particles within this range (*p ≤ 0*.*05*). Finally, particles > 200 nm accounted for 12–15.0% of total isolated EVs for the WT, Sb2000.1 and AmB1000.1 (Fig 2E). The percentage of particles corresponding to this range was significantly increased in MF200.5 (24.0%) when compared to the WT (*p ≤ 0*.*05*).

### Protein diversity and abundance are altered in EVs released by *L*. *infantum* drug-resistant strains

To better understand the functions of EVs, including their potential role in cellular survival in the presence of stress (*e*.*g*. drug resistance), their molecular content (which specifically reflects the phenotype of parent cells) should be analyzed. Since proteins are a major component of EVs and the *Leishmania* proteome is specifically modified in drug-resistant strains, we decided to identify common and unique proteins present in EVs released by the WT and the three resistant strains using untargeted, label-free, shotgun LC–MS/MS proteomics. In this study, we included three independent biological replicates for each strain to minimize the any potential effect derived from EVs’ heterogeneity (S1 Data). We were therefore able to identify common and unique proteins within the same strain, as well as among the different cell lines. Proteomic data was first analyzed using the Scaffold software, which was able to identify a high number of proteins and protein clusters. Subsequent analyses were performed using independent proteins rather than clusters (S1 Data).

**Fig 3:**
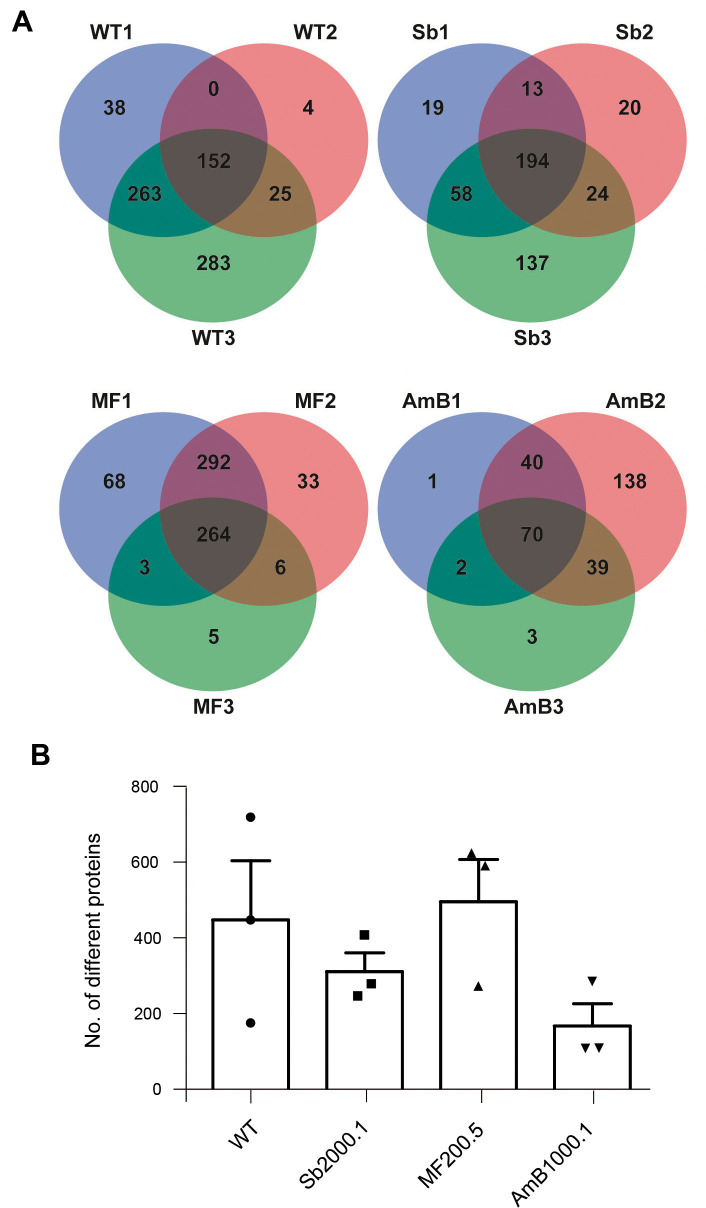
Characterization of EVs’ protein diversity is *L*. *infantum* parasites. **(A)** Venn diagrams summarizing the distribution of the identified proteins by their presence/absence in three biological replicates per strain. **(B)** Total number of different proteins identified in EVs isolated from each strain. Horizontal lines indicate the mean value for each strain (n = 3).

LC–MS/MS analysis identified 152, 194, 264 and 70 proteins shared through three biological replicates for the WT, Sb2000.1, MF200.5, and AmB1000.1 mutants, respectively (Fig 3A). Moreover, we evaluated the total protein diversity of EVs belonging to each strain (Fig 3B). The global proteomic profile of the EVs released by the resistant strains showed significant differences when compared with the WT (S3 Fig). The diversity was variable depending on the replicate, especially for the WT. This variation was less important in the case of the resistant strains, for which two out of three replicates were always very similar. AmB1000.1 EVs showed a tendency to harbour less different proteins than the other three strains (Fig 3B). However, no significant differences were found between the resistant strains and the WT when comparing their average amount of proteins (unpaired t-test, *p > 0*.*05*). Only proteins shared among the three replicates of each strain were retained for subsequent analyses.

Next, using the automated features of Scaffold 4.11, we generated a mean-deviation scatterplot to evaluate the variation of protein abundance in EVs derived from the four strains. (S4 Fig). This analysis shows an estimate of the abundance (mean value of normalized spectral counts) of proteins previously retained for each strain (shared among the three replicates). Proteins recovered from WT (152) and Sb2000.1 (194) EVs were similarly distributed. However, most of the 70 conserved proteins identified for the AmB1000.1 strain (red squares in S4 Fig) were located on the right side of the scatterplot, corresponding to higher abundance levels. High-abundance proteins detected in AmB1000.1 could be masking the detection of low-abundance proteins, leading to a lower total protein diversity. In contrast, proteins identified in MF200.5 EVs were found more frequently on the left section of the scatterplot, which corresponds to lower abundance levels.

### Identification and characterization of *L*. *infantum* EVs’ core proteome

**Fig 4:**
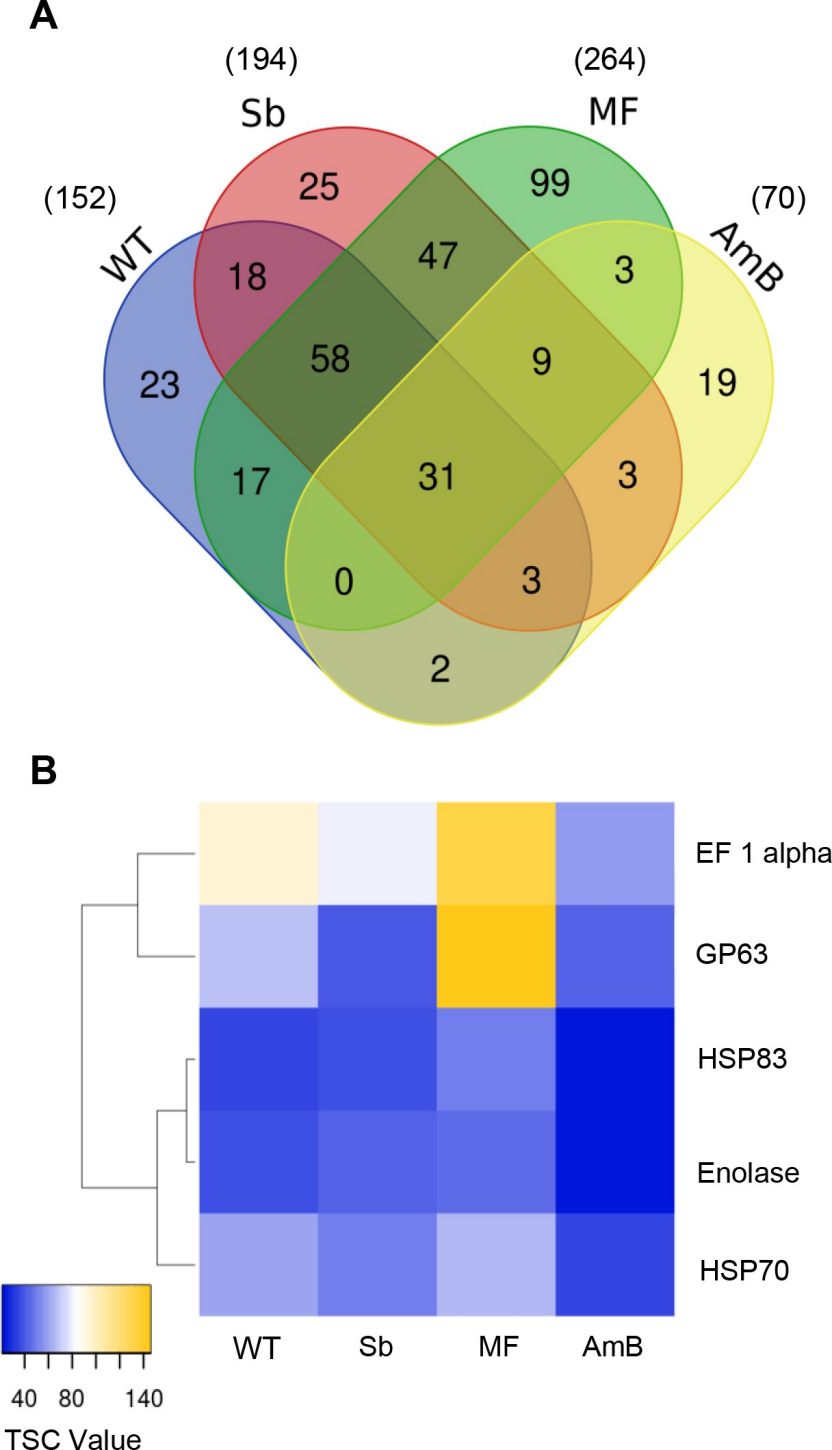
Identification of *L*. *infantum* EVs’ conserved proteins. **(A)** Venn diagram showing all the proteins identified in EVs isolated from *L*. *infantum* WT and drug-resistant strains Sb2000.1, MF200.5 and AmB1000.1. Venn diagram shows common and exclusive proteins in the different groups. The numbers of proteins in the overlapping and non-overlapping areas are indicated (only proteins found in three replicates were included). **(B)** Heatmap of leishmanial EVs’ markers in particles isolated from WT, Sb2000.1, MF200.5 and AmB1000.1 *L*. *infantum* strains. Values correspond to the mean total number of spectra identified for each protein in three replicates.

In order to identify and further characterize the core proteome of *L*. *infantum* EVs (proteins conserved among the WT and the three resistant strains), we relied only on those proteins common to all three biological replicates for each of the four cell lines (WT: 152; Sb2000.1: 194; MF200.5: 264; and AmB1000.1: 70, as depicted in Fig 3B). In this way, the Venn diagram in Fig 4A shows 31 shared proteins between the WT and the drug-resistant mutants (detailed in Table 1), as well as conserved proteins for each strain (WT: 23; Sb2000.1: 25; MF200.5: 99; and AmB1000.1: 19).

Next, and in accordance with MISEV2018 recommendations [55], we proceed to identify and quantify the abundance of specific protein markers previously reported for *Leishmania* EVs [32, 56]. As depicted in the heatmap comparing the WT with the drug-resistant mutants (Fig 4B), vesicles isolated from the four strains contained specific *Leishmania*-EV markers, such as metalloprotease GP63, enolase, heat shock proteins HSP70/HSP83 and elongation factor 1 alpha. Importantly, this heatmap revealed that these markers could be enriched differently in the different strains. In order to validate this hypothesis, we evaluated the levels of enrichment of the 31-shared proteins in the drug-resistant strains, compared with the WT (Table 1). While the levels of most of the conserved proteins remained stable among the different mutants and the WT, our results revealed punctual significant, strain-dependent differences. A fold change > 1.60 was set as the up-regulation threshold, and < 0.60 for down-regulation. As depicted in Table 1, Sb2000.1 EVs showed significantly decreased levels of metalloprotease GP63 (approximately 0.60-fold change depending on the gene involved) and a nucleoside diphosphate kinase (0.57-fold change). These two proteins were also downregulated in EVs released by AmB1000.1 (0.39–0.59 and 0.50-fold, respectively). EVs released by the MF200.5 strain were significantly enriched in tubulin alpha (1.73-fold increase) and tubulin beta chains (2.80-fold increase). While not statistically significant, GP63 showed a tendency toward enrichment in EVs released by MF200.5. Of note, our analyses also revealed the existence of nine proteins common to the three resistant strains but absent in the WT (Fig 4A). These proteins included ribosomal proteins, both 40S and 60S, histone H3, along with transcription factor CBF/NF-Y (S2 Table).

**Fig 5:**
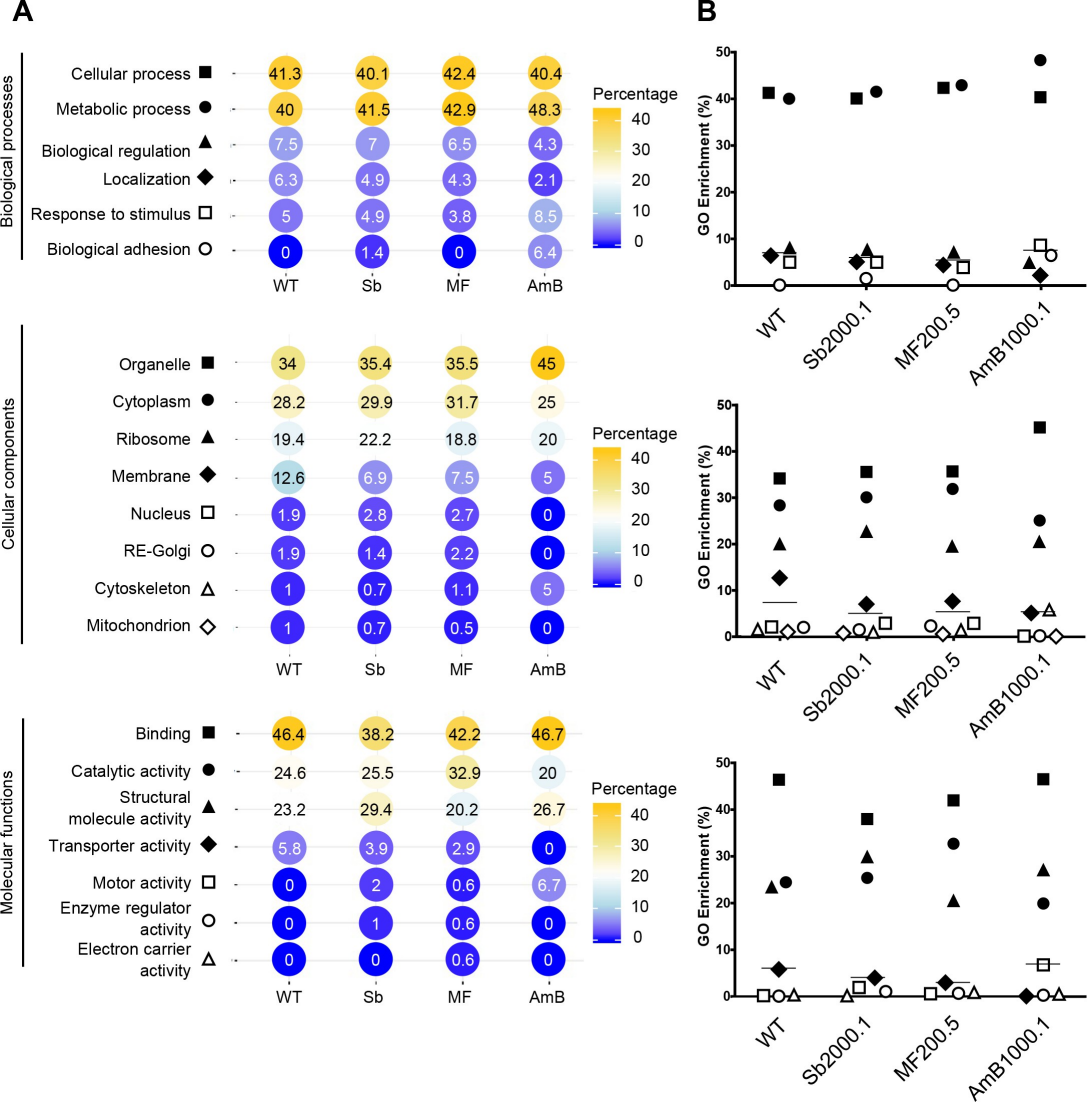
GO analysis of proteins shared by *L*. *infantum* WT and drug-resistant strain EVs. **(A)** Shared proteins among the three replicates of each strain (WT: 152; Sb2000.1: 194; MF200.5: 264; and AmB1000.1: 70 proteins) were annotated with GO terms from UniProt GOA knowledge base using the basic Scaffold version 4.11.0. GO terms were classified according to Biological Process, Cellular Component and Molecular Function, as defined by the GO consortium. **(B)** General distribution of the GO hits identified in (A). Horizontal lines indicate the median values.

Finally, proteins (Table 1) were analyzed in terms of Gene Ontology (GO) annotation, including specific associations related to biological processes, cellular components, and molecular functions (Fig 5). An important limit of our GO analyses in *Leishmania* EVs is the high percentage of unknown proteins present in these particles (approximately 60.0%). Unknown proteins were not included in the GO analysis. For all four strains, EVs proteins were annotated for a wide variety of functions, including cellular and metabolic processes, cytoplasmic and ribosomal components, as well as binding functions (Fig 5A). Among the proteins harboring domains enabling functional assignment, about 40.0% of EVs proteins were functionally ascribed to the metabolism group, approximately 40.0% assigned to the cellular process group, and 4–7.0% to biological regulation processes. The other functional classes (less than 10.0% each) were represented by proteins identified as involved in protein localization, response to stimulus and biological adhesion. Our analysis did not reveal any significant difference between WT and drug-resistant EVs in terms of GO-annotations general distributions (Fig 5B). Nonetheless, we observed specific differences between WT and AmB1000.1 EVs. Proteins found in EVs released by AmB1000.1 were more frequently ascribed to *“biological adhesion”* (biological process), *“cytoskeleton”* (cellular component) and *“motor activity”* (molecular functions) subsets. No protein belonging to AmB1000.1 EVs was annotated within the *“transporter activity”* subcategory (Fig 5A). Of note, EVs released by the three resistant cell lines, but not by the WT, contained variable percentages of proteins annotated for *“motor activity”*. These represented approximately 7.0% of proteins identified in AmB1000.1 EVs. With regard to the cellular compartment of origin of the different proteins, most of them belonged to organelle, cytoplasmic, and ribosomal compartments. As previously reported for EVs, very few proteins were derived from the nucleus, Golgi apparatus, endoplasmic reticulum, or mitochondria [55].

### Drug-resistance leads to differently enriched profiles and unique proteins in *L*. *infantum* EVs

Drug-resistant *Leishmania* parasites modify their general proteome according to the drug that has been involved in the *in vitro* selection or *in vivo* treatment process [34, 35]. Our search for enriched proteins present in EVs specific to each drug-resistance background led to the identification of 23, 25, 99 and 19 proteins for WT, Sb2000.1, MF200.5 and AmB1000.1 EVs, respectively, as shown in the Venn diagram in Fig 4A.

Tables 1–4 depict the complete list of enriched proteins identified in EVs released by the WT and the three drug-resistant strains. The total number of hypothetical conserved proteins and uncharacterized proteins varied for each strain: 0/23 for the WT, 7/25 for Sb2000.1, 11/99 for MF200.5 and 4/19 for AmB1000.1. MF200.5 was the strain showing the greatest diversity, as well as the highest enrichment in terms of total spectrum count (TSC) for the most enriched proteins, which were headed by an ATP-dependent 6-phosphofructokinase (LinJ.29.2620; TSC: 69.3) and a glycosome phosphoenolpyruvate carboxykinase (LinJ.27.1710; TEC: 66.3) (Table 3). The most enriched unique protein in WT EVs was a putative cytoskeleton associated protein CAP5.5 (LinJ.31.0450; TSC: 21.3) (Table 1). An enrichment of different ribosomal proteins (S14 and L37), a hypothetical protein (LinJ.07.0960; TEC: 17.3), a SPRY-domain ubiquitin ligase protein (LinJ.13.1510; TEC: 14.3), and an Ecotin-like protein (LinJ.15.0540; TSC: 10.0) were identified in EVs isolated from Sb2000.1 (Table 2). AmB1000.1 EVs were characterized by a high abundance of a hypothetical conserved protein (LinJ.27.1630; TSC: 37.7), followed by actin (LinJ.04.1250; TSC: 15.3), and a C2 domain protein LinJ.29.0110; TSC: 14.0).

Proteins summarized in Tables 2–5 constitute the conserved proteomic profile of EVs released by the different strains studied. These proteins were shared by the three replicates of one specific strain, but were not shared by the three replicates of any of the other strains. While these profiles point to drug-dependent enrichments in the different strains, this data does not pinpoint unique proteins that could be proposed as biomarkers. To determine if some of the enriched proteins could be considered strain-specific (absent in all the replicates of the rest of the strains), we performed a cross-correlation comparison (S5 Fig) between excluded and common pools of proteins previously retained to generate the EV profiles (reported in Fig 3A). This analysis revealed 4 unique enriched proteins in EVs released by WT (Table 2) and MF200.5 (Table 4) strains, and 13 unique proteins in the AmB1000.1 strain (Table 5).

Previous studies have reported that unconventional secretion is a key feature of EVs’ proteins in *Leishmania* [57]. To validate this among the unique, enriched proteins identified in our four strains, we used SecretomeP 2.0 proteomic tool [52] for identification of non-classical protein secretion. Of note, this server integrates SignalP5.0 for further confirmation of the presence of signal peptides [53]. Our analyses showed that approximately 26.0% of WT-unique proteins are unconventionally secreted (Table 2; NN-score > 0.600 and absence of signal peptide). This value was slightly lower for the three drug-resistant strains, with values of 20.0%, 21.8%, and 15.8% for Sb2000.1, MF200.5, and AmB1000.1 EVs, respectively (Tables 3–5). On the other hand, according to SignalP prediction, 13.0% of the unique proteins identified in WT EVs have signal peptides (Table 2). Regarding drug-resistant strains, 20.0% of Sb2000.1 and 7.9% of MF200.5 EVs unique proteins were predicted with signal peptides (Tables 3 and 4). No signal peptide was identified among of the proteins identified in AmB1000.1 EVs (Table 5). As EVs usually include various membrane proteins, we manually assessed the presence of membrane/transmembrane proteins using UniprotKB [48]. As summarized in Tables 2–5, the number of unique, enriched membrane/transmembrane proteins for each strain was very variable: 9 (39.1%) for the WT, 5 (20.0%) for Sb2000.1, 4 (4.0%) for MF200.5 and 2 (10.5%) for AmB1000.1.

## Discussion

Different parasite molecular modifications, such as ABC transporters, membrane composition changes or oxidative stress, among others, can contribute both directly and indirectly to the phenomena of drug resistance [35, 46, 58]. Moreover, it has been shown that *Leishmania* is able to actively alter macrophage and neutrophil environments to resist current antileishmanial agents [59]. Recently, different drug-resistant field isolates have shown either efficient survival inside the insect vector or increased infectivity in the mammalian host [60–64]. This highlights the importance of not only parasite-to-parasite communication, but also a more complex host-parasite and vector-parasite environment. EVs have become known as an integral part of the parasite’s infectious life cycle as powerful cell-cell communication mediators. *Leishmania* infection, parasite survival (both inside the vector and the host), as well as the resulting clinical manifestations, are highly influenced by EVs [21–23, 29, 30, 65]. While traditional studies have focused on the survival of drug-resistant parasites in the host during treatment (amastigote form), the impact of drug resistance on the survival of the parasites inside the sand-fly vector (promastigote form) and the first moments of infection has recently begun to be explored. The latter could have a major impact on the spread and perpetuation of drug-resistant *Leishmania* populations [60–62, 66]. To better understand the potential roles of EVs in both the development of *Leishmania* parasites and the spread of drug-resistant strains, we have isolated and characterized the EVs released by the promastigote form of three drug-resistant *L*. *infantum* strains, as well as their WT counterpart. Our results showed, for the first time, that EV quantities, morphology, distribution, and protein cargo are altered in drug-resistant *Leishmania* parasites. Moreover, these changes appear to be specific to the antileishmanial drug involved in the resistant phenotype.

First, we investigated the impact of drug resistance on the production and distribution of EVs, which could lead to these extracellular elements serving different biological functions. While all four strains were able to produce particles compatible with EVs, parasites resistant to both miltefosine (MF200.5) and amphotericin B (AmB1000.1) produced larger EV subpopulations with altered morphology, especially in MF200.5, as shown by TEM and nanoparticle-tracking analysis. It is known that resistance to miltefosine and amphotericin B in *Leishmania* can lead to drastic changes in different lipids species, predominantly in sterols and phospholipids, some of which are enriched with cyclopropanated fatty acids, as well as to an increase in inositolphosphoceramide species [46, 67]. Consequently, MF- and AmB-resistant parasites tend to show a significant increase in membrane rigidity [67, 68]. Similarly, AmB1000.1 parasites are greatly depleted in ergosterol and 5-dehydroepisterol, but highly enriched in 4-methyl-8,24-cholestadienol. Likewise, MF200.5 cells contain strikingly low levels of ergosterol, with episterol as predominant sterol [46]. Due to the generation of exosomes from late endosomes [38], these vesicles are highly enriched in sterols (up to 43%) in eukaryotic cells [69]. As such, alterations in sterols, and thus membrane fluidity, could be responsible for the marked morphological changes observed in EVs from the MF200.5 strain. Specific EV-targeting lipid analysis should be conducted to further support this hypothesis. Nanoparticle-tracking analyses revealed a significant increase in the modal particle size of MF200.5 and AmB.1000.1 EVs. This is in correlation with previous TEM studies in which *L*. *infantum* parasites were stimulated with G418 (geneticin), leading to an increased release of larger vesicles in response to death-inducing stimuli [70]. Likewise, gentamicin-resistant pathogenic *E*. *coli* increased the modal size of their outer membrane vesicles (OMVs) when exposed to high concentrations of gentamicin [71]. Similarly, a recent study showed that β-lactam antibiotic-resistant *E*. *coli* produced significantly larger OMVs than its sensitive counterpart [72]. Particle-size distribution analyses revealed significant differences between the four strains, as well as important levels of heterogeneity, which is in accordance with previous reports involving different eukaryotic-cell lines [73]. MF200.5 and AmB1000.1 showed significantly increased amounts of particles within the ranges 100–200 and >200 nm, respectively. Among biomolecules, sterols, especially cholesterol, play a major role in the formation of EVs. As such, the above-mentioned modifications in sterol species in the MF200.5 and AmB1000.1 mutants [46] could potentially be inducing changes in the formation and release of small EVs (normally associated with exosomes). Moreover, while there is a growing body of knowledge suggesting a pathway analogous to that of the mammalian ESCRT-dependent pathway previously reported, the precise mechanism in *Leishmania* has yet to be fully elucidated (reviewed in [65]). It is therefore possible that this pathway, as well as different molecular sorting mechanisms, have been altered in drug-resistant mutants. For example, EVs released by Sb2000.1 and AmB1000.1 showed significantly reduced metalloprotease GP63 content, which has been shown to participate in the sorting of protein content of *Leishmania* exosomes [30].

During their biogenesis, EVs are hypothesized to selectively capture cell-specific proteins that may then become a part of the EVs “molecular signature”. However, the mechanism of such selective packaging remains unknown [74]. To investigate the proteomic “molecular signature” of *Leishmania* EVs, we first evaluated the protein diversity found in the EVs released by the WT and three drug-resistant strains. No significant differences between strains were observed in terms of number of different proteins. This could be explained by the fact that proteins in EVs account for more than half of the total secretion of parasite proteins [75], and EVs, due to their size, have a limited space to enclose large quantities. Moreover, *Leishmania* genomic organization and control leads to great individual parasite-to-parasite variations resulting in mosaic aneuploidy [76, 77], which ultimately modulates protein expression [35]. Due to the specialization of drug-resistant parasites required for their survival (compared with the WT), a lower parasite-to-parasite variability in these strains could be expected, which, coupled with the larger size of drug-resistant EVs, could lead to lower variability within the same strain when compared with the WT. MF200.5 EVs showed a tendency to contain a more diverse EV protein population, which could be facilitated by their larger size (when compared to the other strains). On the other hand, despite its EVs being significantly larger than those of the WT, the AmB1000.1 strain showed the lowest EV protein diversity. This lower diversity was accompanied by an increase in protein abundance. That said, high-abundance proteins could be masking the detection of low-abundance proteins in AmB1000.1, leading to a lower total protein diversity [78]. We also identified the presence of several conserved markers in the EVs isolated from the three replicates of the four strains. These corresponded to EV-specific markers previously reported for *Leishmania* and other eukaryotic cells [32, 55, 56]. The zinc-metalloprotease GP63, in addition to the protein sorting functions discussed above [30], is a virulence factor that greatly influences host cell signaling mechanisms and related functions [79]. This analysis allowed us to identify a number of correlations between EVs’ composition and the whole-cell proteome of the drug-resistant strains, demonstrating that EVs could reflect the drug-resistance background of their parental cell line. In this way, previous proteomic studies targeting drug-resistant promastigotes reported a downregulation of GP63 in both Sb2000.1- and AmB1000.1-resistant parasites (Sb/WT ratio: -1.91 and AmB/WT ratio: -4.14, respectively) [42, 44]. In our experiments, GP63 was significantly underrepresented in Sb2000.1 and AmB1000.1 EVs. Likewise, proteins enriched in drug-resistant EVs, such as 14-3-3 Protein-like protein (in AmB1000.1) or Tryparedoxin peroxidase (in Sb2000.1), were also enriched in whole-parasite preparations (AmB/WT ratio: +1.98 and Sb/WT ratio: +1.38, respectively) [42, 44]. Another example is a kinesin coded by the gene LinJ.16.1550, which was enriched 2.75-fold in Sb-resistant parasites compared with their WT counterpart in SILAC experiments [42]. Similarly, all three replicates of EVs released by Sb2000.1, but none of the other investigated strains, showed the presence of this specific protein. This is the first time it has been demonstrated that the proteomic signature profile of EVs–and thus protein sorting–of *Leishmania* parasites could vary according to their drug-resistance profile, as previously observed for cancer cells and bacteria [38–40].

The mechanisms of drug-resistance in *Leishmania* parasites are far from completely elucidated, and some may be non-specific adaptations that provide a general fitness gain allowing the parasite to survive in stressful conditions. In the search for non-specific mechanisms of drug resistance, we identified an interesting subset of nine EVs’ proteins enriched in the three replicates of the three drug-resistant strains, but absent in the WT. These proteins included histone H3, core histone-like transcription factor (CBF/NF-Y), and different ribosomal proteins. Histones play an important role in DNA packaging, transcription and gene regulation in the parasite, which also includes drug-resistant genes. This is in accordance with previous studies in *L*. *donovani*, in which Sb-resistant strains isolated from patients showed an overexpression of histones, highlighting the potential role of these proteins as traits of drug resistance [80]. While it is well documented that *Leishmania* EVs are effective modulators of early macrophage and host inflammatory responses [29–31], the exact mechanisms behind this modulation are yet to be characterized. Strikingly, a recent study discovered a new virulence mechanism in *L*. *major*, by which *Leishmania* H3, one of the histones only present in our drug-resistant EVs is secreted by the parasite and forms a nucleosome with the human histones in the host chromatin during infection. This leads to a relaxed conformation of chromatin that impacts gene expression pattern, facilitating survival of the parasite [81]. Transcription factor CBF/NF-Y has been shown to be regulated in different cell types when submitted to different types of stress, such as mechanical stress, ER stress, and DNA damage-related stress, among others [82]. Its presence in EVs derived from drug-resistant parasites would reflect general adaptation mechanisms against drug-generated stress. Moreover, drug-resistant EVs, but not naïve EVs, would have the potential to modulate transcription in recipient *Leishmania* parasites with the potential to mediate different downstream pathways. Similarly, it has recently been shown that cancer cells may exploit exosomes to confer transcriptome reprogramming that leads to cancer-associated pathologies [83]. Drug-resistant EVs were also enriched in ribosomal proteins. Similarly, several ribosomal proteins were upregulated in Sb-resistant *L*. *donovani* field isolates, decreasing the sensitivity of the parasites to Sb, MF, and paromomycin by increasing the proliferation of parasites [84].

As previously discussed, EVs capture cell-specific proteins during their formation and release. For this reason, we were interested in what EVs could potentially tell us about the biology, immunopathology, and drug-resistance mechanisms deployed by each strain. Recent evidence points to a possible correlation between Sb-resistance and virulence in *Leishmania* field isolates [66]. Interestingly, among the 25 enriched proteins present in Sb2000.1 EVs, one of the most upregulated proteins was a SPRY domain-containing ubiquitin ligase. The SPRY domain-containing SOCS box protein 2 was shown to recruit an E3 ubiquitin ligase complex to polyubiquitinate iNOS, targeting it for proteasomal degradation and therefore reducing macrophage killing of *Leishmania* parasites [85]. Moreover, Sb2000.1 EVs contained two proteasome regulatory subunits (an ATPase and a non-ATPase). This suggests that Sb2000.1-derived EVs could be involved in the modulation of redox control and protein degradation of recipient parasites, leading to increased tolerance to stress-inducing drugs as well as to the intracellular environment. Another important protein highly enriched in Sb2000.1 EVs was an ecotin-like protein, which has an important role in the inhibition of peptidases and promotion of *Leishmania* survival inside the sand fly vector. In addition, this protein modulates parasite differentiation and increases macrophage internalization due to the upregulation of phagocytosis by a mechanism dependent on serine peptidase activity [86, 87]. With respect to the MF200.5 strain, released EVs were enriched in glycolytic (*e*.*g*. ATP-dependent 6-phosphofructokinase and glycosomal phosphoenol pyruvate carboxykinase) and lipid-related (*e*.*g*. inosine 5’-monophosphate dehydrogenase and sterol 14*α*-demethylase) pathways, reflecting MF’s mode of action and mechanisms of drug resistance previously described in whole-parasite studies [46, 59]. MF200.5 EVs carried two ATP-dependent RNA helicases. One of them was a member of the DEAD-box family, which has been shown to play a central role in preventing ROS-mediated damage and in maintaining mitochondrial protein quality control in *Leishmania* [88]. This would facilitate the survival of parasites against mitochondrial MF-induced ROS. In addition, several partners of the DEAD-box helicase [88] were also found in MF200.5 EVs. For example, the HS70-related protein-1 mitochondrial precursor (HSPA9B) has been recently linked to miltefosine resistance and response to oxidative stress in *L*. *donovani* [89]. In addition to GP63, several other virulence factors, such as disulfide-isomerase and cysteine peptidase B (CPB) proteins, were found in MF200.5 EVs, further confirming that *Leishmania* EVs play a major role as virulence factors [21, 65]. This also raises the concern about a potentially increased virulence induced by EVs secreted by drug-resistant parasites. Last, analysis of AmB1000.1 EVs led to the identification of 19 enriched proteins. Among these, we identified a trypanothione synthetase (TryS) and an ATP-binding cassette protein (MRPA/P-gpA), the latter of which was also present in 2/3 replicates of Sb2000.1. Resistance to AmB in *Leishmania* has been associated with increased expression of enzymes involved in thiol metabolism. Equbal and collaborators showed that TryS mRNA is upregulated in AmB-resistant *L*. *donovani* field isolates when compared with sensitive strains [90]. Increased levels in the thiol pathway could also explain the presence of MRPA, which is implicated in the sequestration of thiol conjugates near the flagellar pocket (a specific site for exosome accumulation and secretion in *Leishmania* [56, 57]), and subsequent exocytosis outside the cell [91]. Moreover, in relation to MRPA, there is considerable evidence that P-gp and other MDR transporters can be transmitted from drug-resistant to drug-sensitive tumor cells by exosomes *in vivo* and *in vitro* [92]. Ongoing research will shed light on the potential occurrence of similar mechanisms in *Leishmania* parasites.

The final question we broached was the potential use of unique proteins present in EVs as biomarkers of drug resistance. Since *Leishmania* EVs are highly enriched within the sand fly and are co-egested with the parasite during the insect’s bite [56], drug-specific biomarkers would represent a major epidemiological tool for the evaluation of drug-resistant *Leishmania* populations, as well as increase our knowledge on how drug-resistant parasites adapt to mammalian and insect hosts in order to guarantee survival. Our results demonstrate that different proteomic-signature profiles can be obtained depending on the drug-resistance background of the parental cell. Moreover, we were able to identify two sets of four unique proteins present only in EVs released by the WT and MF200.5, as well as 13 in AmB1000.1 particles. While our study represents the first step towards the discovery of EV-based drug-resistance biomarkers, our work presents two limitations that should be addressed in future studies: future analyses should target larger sets of strains and replicates in order to reduce the observed variability and improve the robustness of potential biomarkers. Furthermore, evaluation and comparison of EV production and composition directly from resistant field strains is required.

In conclusion, we have conducted the first comparative characterization of *Leishmania* EVs in the context of drug resistance. We have analyzed the EVs of three different *L*. *infantum* strains resistant to antimony, miltefosine and amphotericin B, respectively, as well as their WT counterpart. Our results showed for the first time that drug-resistance mechanisms can induce changes in the morphology, size, and distribution of EVs in *Leishmania*. Next, we identified the core proteome of EVs conserved in both sensitive and drug-resistant backgrounds. Moreover, we obtained the first snapshot of the enriched and unique proteins in EVs released by each drug-resistant strain. Additionally, among these enriched EVs proteins, we identified several virulence factors, transcription factors, as well as proteins coded by drug-resistance genes. This selective sorting of cargo could facilitate the survival of drug-resistant parasites, and potentially sensitive parasites in contact with drug-resistant strains, to a plethora of stressful situations including drug pressure, initial moments of infection, adaptation to the sand-fly vector, etc. Future studies may explore all of these aspects and shed light on how EVs, especially those released by drug-resistant parasites, contribute to the survival of *Leishmania* during its life cycle.

## Supporting information

S1 Fig EVs released by drug-resistant *L. infantum* parasites show altered particle profiles and secretory capacity. *L*. *infantum* WT and drug-resistant strains were compared in terms of number of particles per category (size) between 0 nm and 500 nm using NTA. **(A-B)** Two representative images of three experiments is shown (experiment 1 is shown in Fig 2A).
(PDF)

S2 Fig EVs released by drug-resistant *L. infantum* parasites show altered particle distributions. Particle distribution analysis corresponding to the range of 0 to 500 nm. Horizontal lines indicate the median value for each particle size (n = 3). Whiskers correspond to minimum and maximum values. Differences were statistically evaluated using Shapiro-Wilk Test for normality, followed by Kruskal-Wallis rank sum test (****p ≤ 0.*0001*). Nemenyi post-hoc test pinpointed significant differences between WT and Sb2000.1 EVs distributions (*p ≤ 0*.*0001*), as well as between Sb2000.1 and the other two resistant strains (**p ≤ 0.*01* for Sb2000.1 *vs*. MF200.5; ****p ≤ 0.*0001* for Sb2000.1 *vs*. AmB1000.1).
(PDF)

S3 Fig Volcano plot of proteins found in *L. infantum* EVs. The volcano plot shows the intensity of protein expression between EVs from Sb2000.1 **(A)**, MF200.5 **(B)** and AmB1000.1 **(C)** cells and EVs from *L*. *infantum* WT cells. The horizontal axis represents the log2 fold change and the vertical axis represents −log10 (Fisher exact test, P value).
(PDF)

S4 Fig Study of the potential impact of protein diversity in protein abundance. Standard deviation scatterplot depicting the mean and standard deviation values of the abundance (normalized total spectra) of each EVs protein transformed into base-10 logarithms for WT, Sb2000.1, MF200.5 and AmB1000.1 (n = 3). X-axis: Log_10_ of the mean value of the estimated protein abundance across those proteins retained for each strain (shared among the three replicates).Y-axis: Log_10_ of standard deviation of the estimated protein abundance computed across those proteins retained for each strain (shared among the three replicates).
(PDF)

S5 Fig Cross-correlation between excluded and common pools of proteins used for identifying enriched proteins in EVs. Enriched proteins common to the three replicates of EVs isolated from WT **(A)**, Sb2000.1 **(B)**, MF200.5 **(C)** and AmB1000.1 **(D)** and their relationship with the pool of proteins excluded because they were not conserved through the replicates of the other strains.
(TIF)

S1 Table Drug sensitivity profiles of the different strains used in this study. EC_50_ values were calculated from the dose-response curves after performing a nonlinear fitting with the GraphPad 8.0 software program. An average of three independent biological replicates is shown ± standard error of the mean.
(PDF)

S2 Table Proteins common to EVs released by the three drug-resistant strains but not by the WT. (PDF)

S1 Data Raw proteomic data used in this study. Total Spectra of *Leishmania* EVs using *Leishmania infantum* database.
(XLSX)
