# Supplementary figures and images for: Unravelling the proteomic signature of extracellular vesicles released by drug-resistant Leishmania infantum parasites
Source: PLoS Negl Trop Dis. 2020 Jul 6;14(7):e0008439. doi: 10.1371/journal.pntd.0008439 (PMC7365475; doi:10.1371/journal.pntd.0008439)

**A**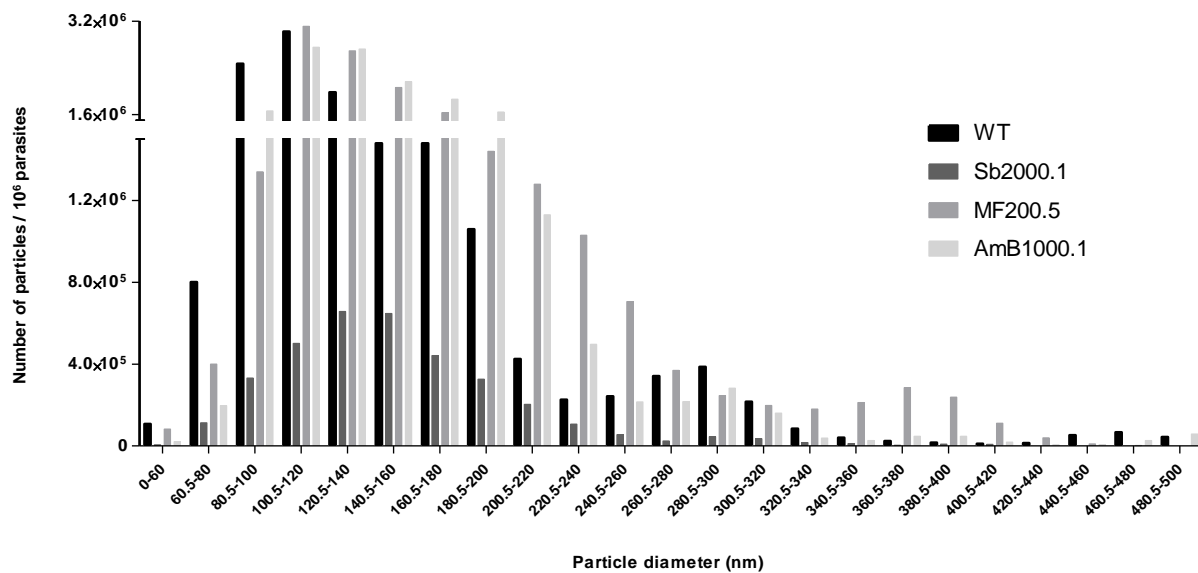**B**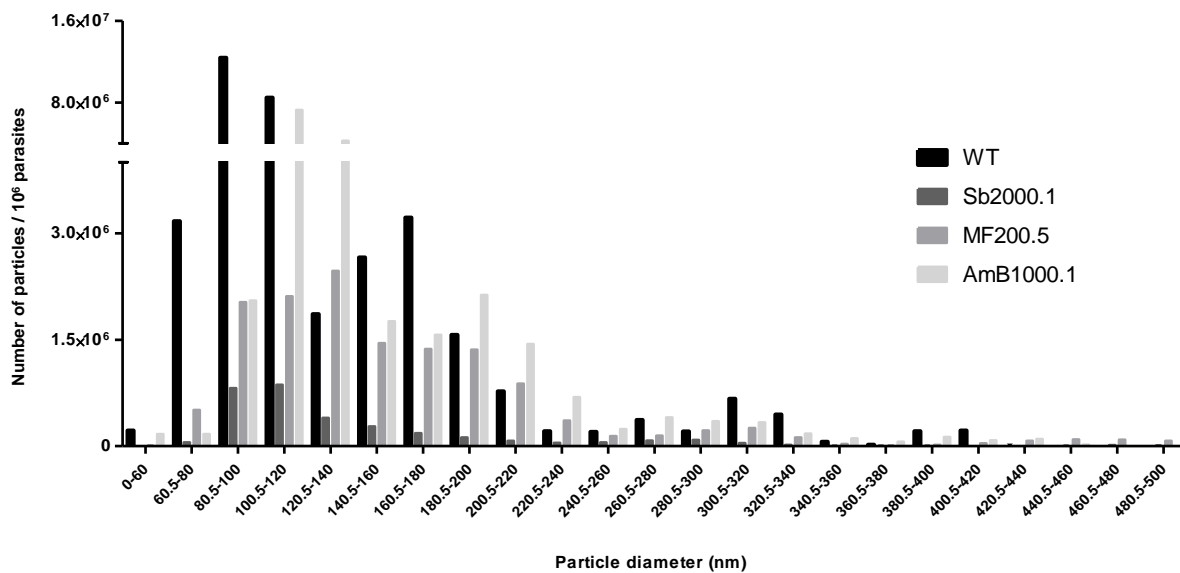

Supplement: S1 Fig — L. infantum WT and drug-resistant strains were compared in terms of number of particles per category (size) between 0 nm and 500 nm using NTA. (A-B) Two representative images of three experiments is shown (experiment 1 is shown in Fig 2A). (PDF) [file pntd.0008439.s001.pdf]

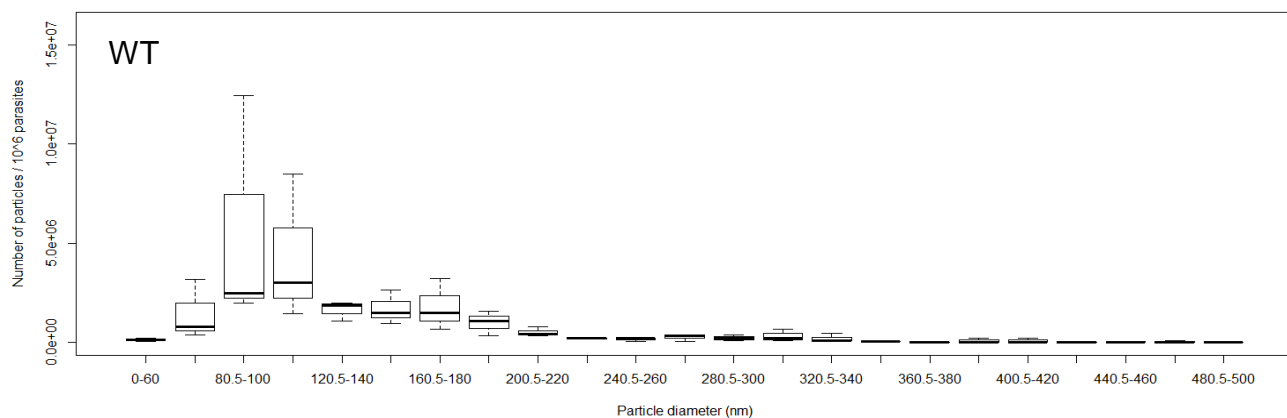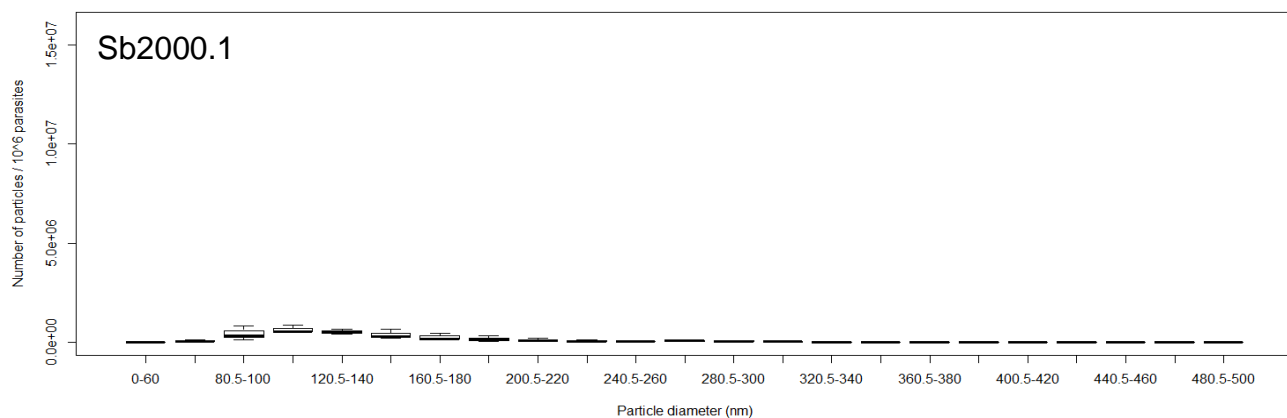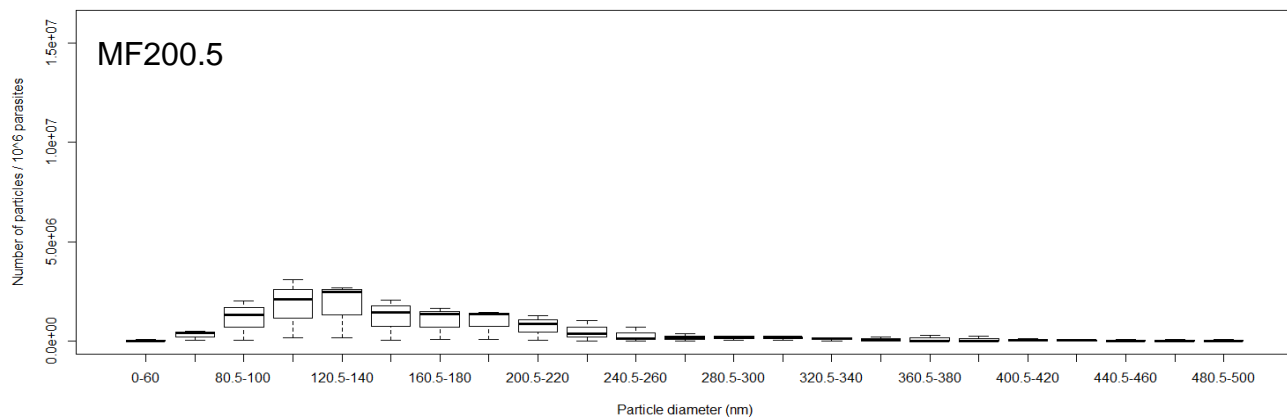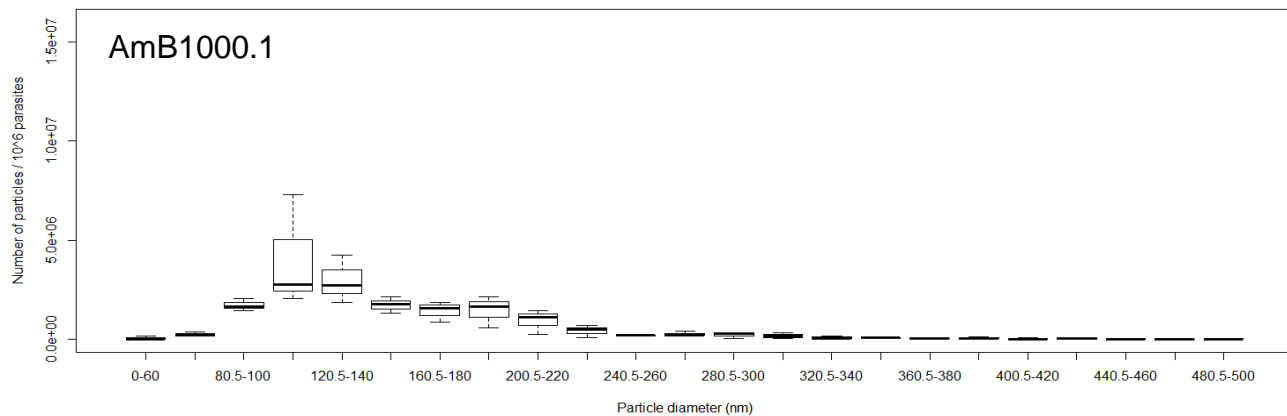

Supplement: S2 Fig — Particle distribution analysis corresponding to the range of 0 to 500 nm. Horizontal lines indicate the median value for each particle size (n = 3). Whiskers correspond to minimum and maximum values. Differences were statistically evaluated using Shapiro-Wilk Test for normality, followed by Kruskal-Wallis rank sum test (****p ≤ 0.0001). Nemenyi post-hoc test pinpointed significant differences between WT and Sb2000.1 EVs distributions (p ≤ 0.0001), as well as between Sb2000.1 and the other two resistant strains (**p ≤ 0.01 for Sb2000.1 vs. MF200.5; ****p ≤ 0.0001 for Sb2000.1 vs. AmB1000.1). (PDF) [file pntd.0008439.s002.pdf]

A

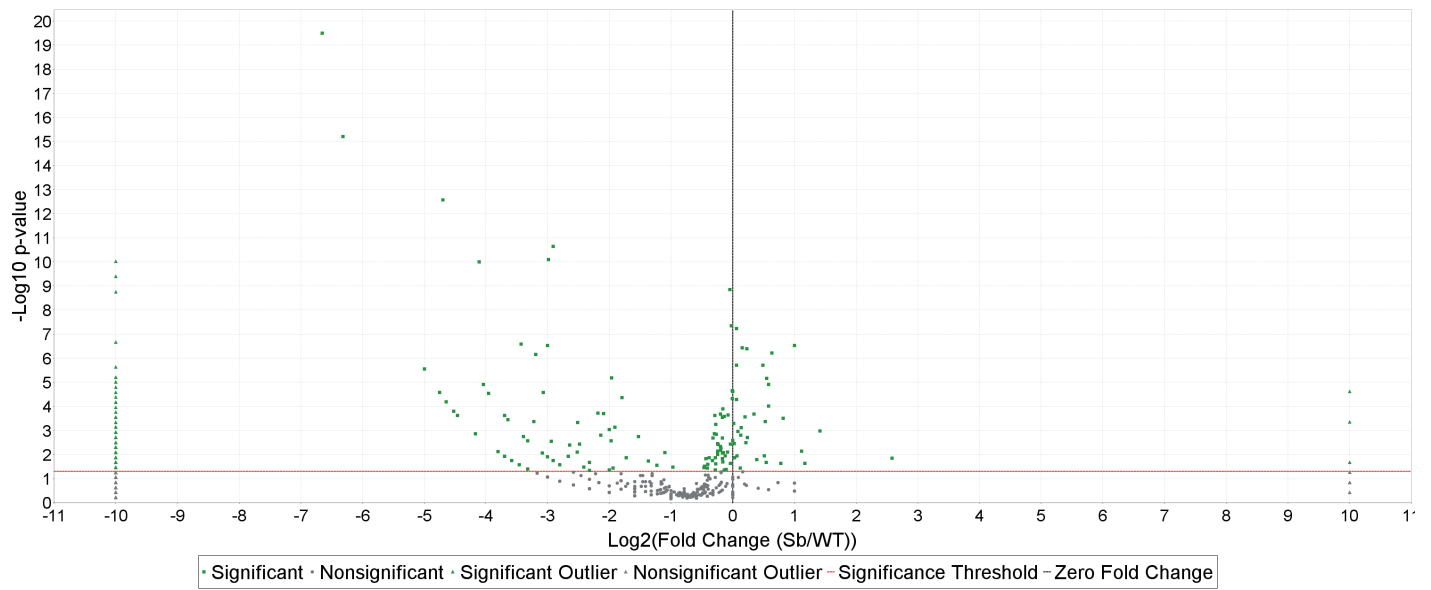

B

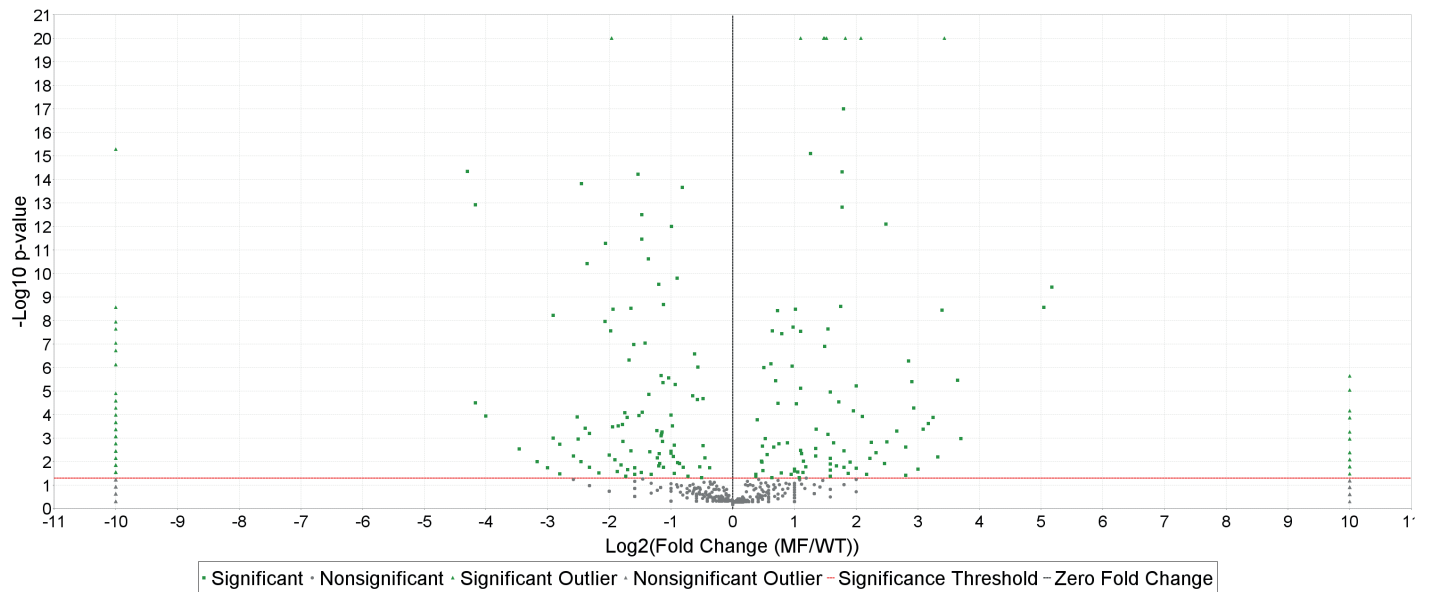

C

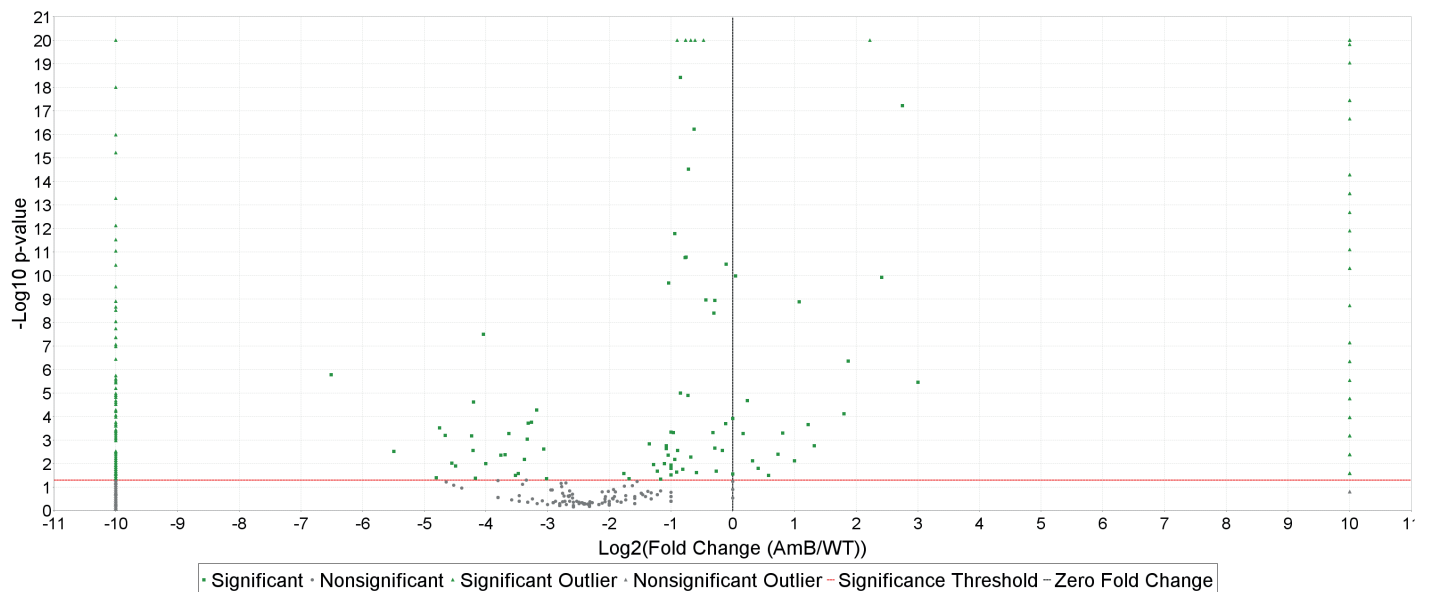

Supplement: S3 Fig — The volcano plot shows the intensity of protein expression between EVs from Sb2000.1 (A), MF200.5 (B) and AmB1000.1 (C) cells and EVs from L. infantum WT cells. The horizontal axis represents the log2 fold change and the vertical axis represents −log10 (Fisher exact test, P value). (PDF) [file pntd.0008439.s003.pdf]

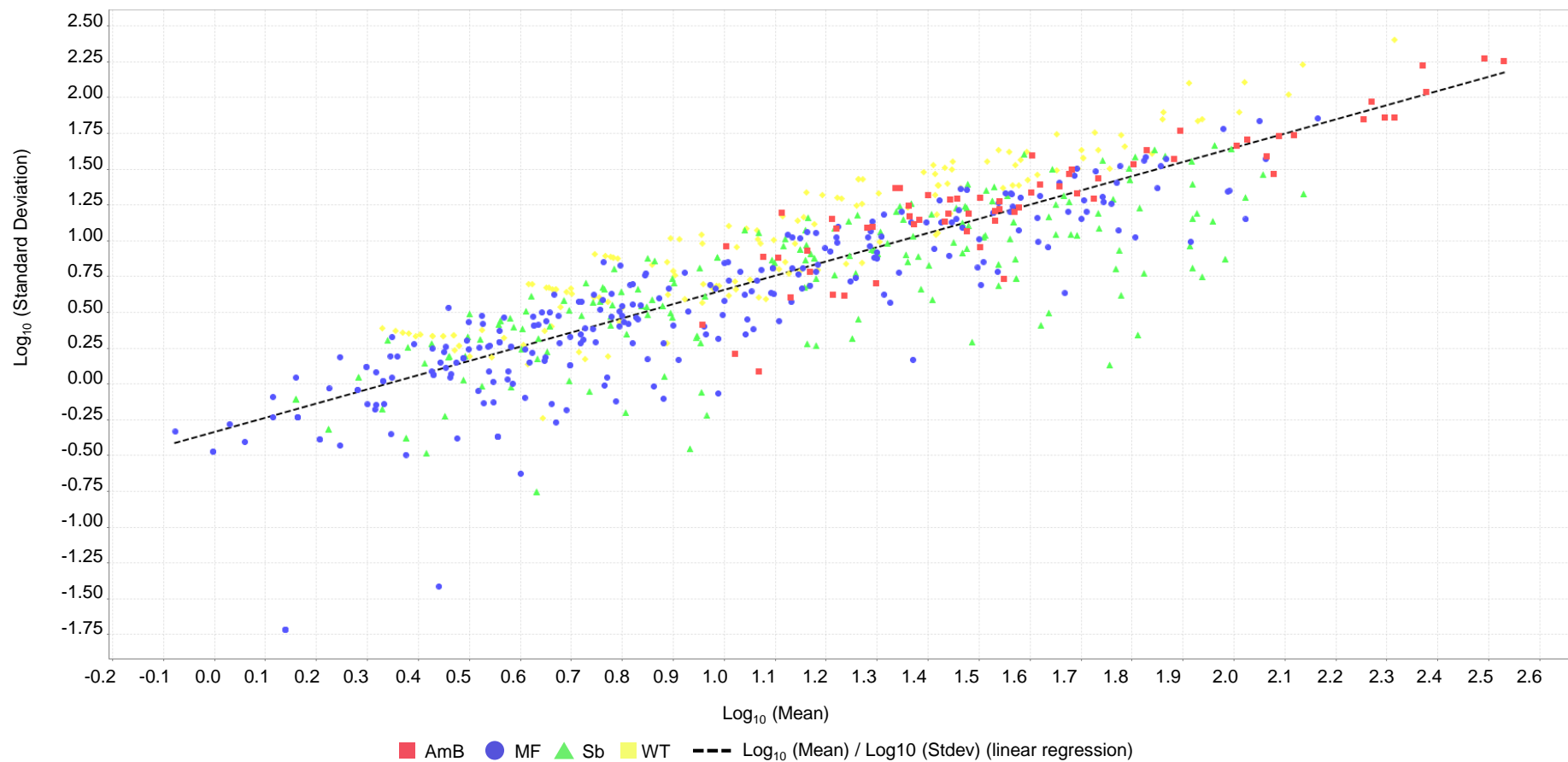

Supplement: S4 Fig — Standard deviation scatterplot depicting the mean and standard deviation values of the abundance (normalized total spectra) of each EVs protein transformed into base-10 logarithms for WT, Sb2000.1, MF200.5 and AmB1000.1 (n = 3). X-axis: Log10 of the mean value of the estimated protein abundance across those proteins retained for each strain (shared among the three replicates).Y-axis: Log10 of standard deviation of the estimated protein abundance computed across those proteins retained for each strain (shared among the three replicates). (PDF) [file pntd.0008439.s004.pdf]

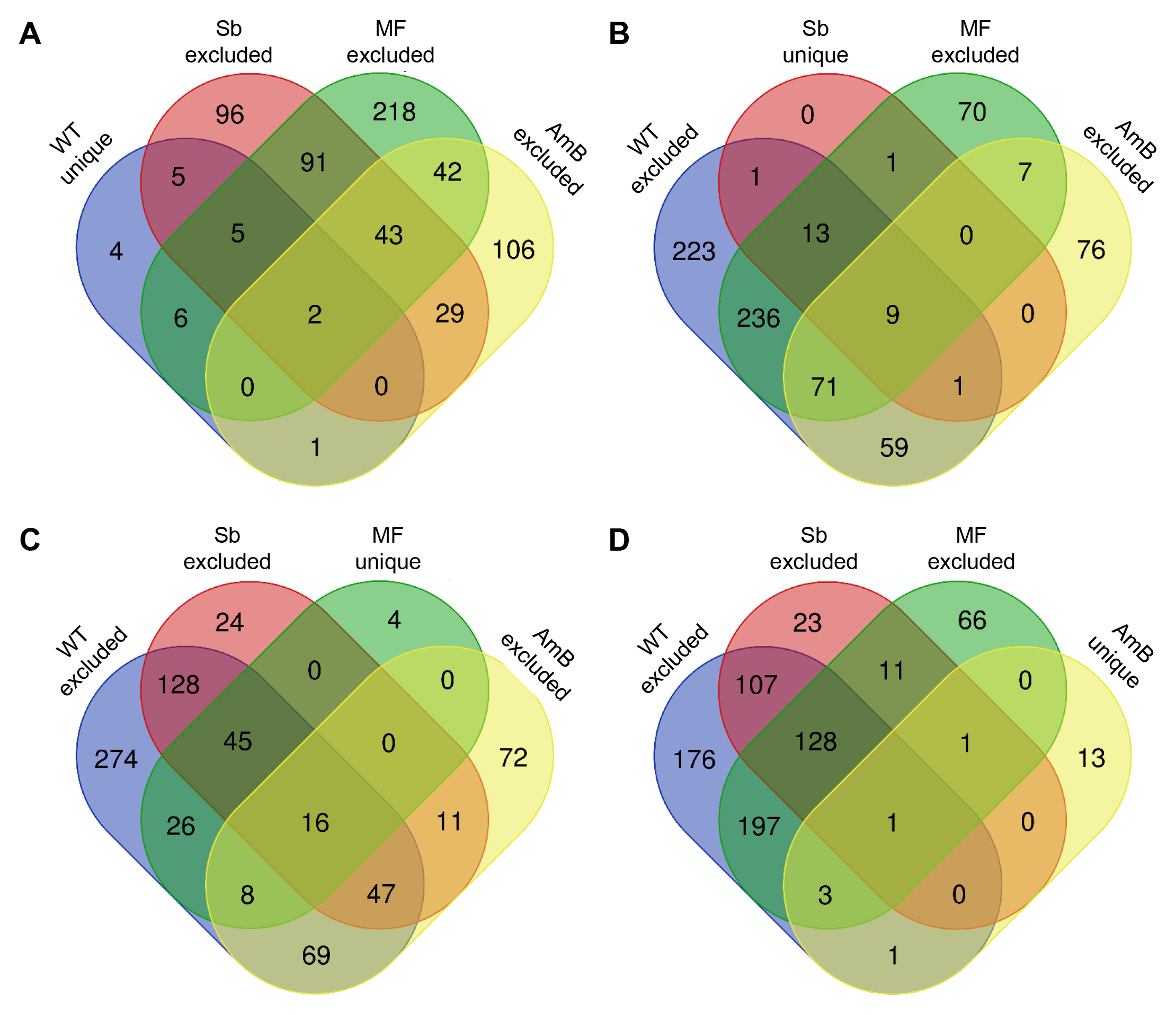

Supplement: S5 Fig — Enriched proteins common to the three replicates of EVs isolated from WT (A), Sb2000.1 (B), MF200.5 (C) and AmB1000.1 (D) and their relationship with the pool of proteins excluded because they were not conserved through the replicates of the other strains. (TIF) [file pntd.0008439.s005.tif]
