# Supplementary material for: Unravelling the proteomic signature of extracellular vesicles released by drug-resistant Leishmania infantum parasites
Source: PLoS Negl Trop Dis. 2020 Jul 6;14(7):e0008439. doi: 10.1371/journal.pntd.0008439 (PMC7365475; doi:10.1371/journal.pntd.0008439)
Supplement: S1 Table — EC50 values were calculated from the dose-response curves after performing a nonlinear fitting with the GraphPad 8.0 software program. An average of three independent biological replicates is shown ± standard error of the mean. (PDF) [file pntd.0008439.s006.pdf]

S1 Table. Drug sensitivity profiles of the different strains used in this study. EC_{50} values were calculated from the dose-response curves after performing a nonlinear fitting with the GraphPad 8.0 software program. An average of three independent biological replicates is shown ± standard error of the mean.

	**Antimony (µM)**	**Miltefosine (µM)**	**Amphotericin B (nM)**
**WT**	77.81 ± 6.80	12.76 ± 3.40	50.85 ± 8.24
**Sb2000.1**	> 300	9.35 ± 1.20	60.68 ± 10.20
**MF200.5**	85.12 ± 10.25	> 200	48.50 ± 10.05
**AmB1000.1**	80.98 ± 7.90	30.80 ± 2.11	> 1,000
