# Supplementary material for: Unravelling the proteomic signature of extracellular vesicles released by drug-resistant Leishmania infantum parasites
Source: PLoS Negl Trop Dis. 2020 Jul 6;14(7):e0008439. doi: 10.1371/journal.pntd.0008439 (PMC7365475; doi:10.1371/journal.pntd.0008439)
Supplement: S2 Table — (PDF) [file pntd.0008439.s007.pdf]

S2 Table. Proteins common to the three drug-resistant strains.

**Gene ID** (Uniprot)	**Protein** (Uniprot)	**Sb2000.1 exosomes**	**MF200.5 exosomes**	**AmB1000.1 exosomes**
LINJ_21_2150	40S ribosomal protein S6	56.7	57.3	14.7
LINJ_29_2570	60S ribosomal protein L13 putative	60.0	52.3	15.7
LINJ_15_0220	60S ribosomal protein L13a putative	55.7	73.3	7.3
LINJ_22_1370	60S ribosomal protein L14 putative	25.7	46.7	8.3
LINJ_24_0040	60S ribosomal protein L22p/L17e putative	34.7	19.7	11.3
LINJ_24_2140	60S ribosomal protein L26 putative	17.7	9.7	4.0
LINJ_07_0550	60S ribosomal protein L7a putative	30.7	35.3	8.7
LINJ_16_0600	Core histone-like transcription factor (CBF/NF-Y)	6.7	8.0	2.7
LINJ_10_1050	Histone H3 putative	7.0	5.0	2.7

Numbers correspond to the Mean Total Spectrum Count obtained with the LC–MS/MS analyses (See S1 Data for more details)
